# Systematic Analysis of the Differential Effects of Red Meat on Colorectal Cancer Risks: A Meta-Analytic Approach

**DOI:** 10.1007/s12029-025-01247-3

**Published:** 2025-08-07

**Authors:** Jun Yu Woon, Gihani Vidanapathirana, Alfred K. Lam, Vinod Gopalan

**Affiliations:** 1 School of Medicine and Dentistry, Griffith University, Gold Coast Campus, Southport, Gold Coast, QLD 4222 Australia; 2 Department of Medical Laboratory Science, Faculty of Allied Health Sciences, University of Peradeniya, Peradeniya, Sri Lanka; 3 Pathology Queensland, Gold Coast University Hospital, Southport, QLD 4215 Australia

**Keywords:** Colorectal cancer, Red meat, Beef, Pork, Lamb, Dietary risk factors

## Abstract

**Objectives:**

Colorectal cancer (CRC) is the third most common cancer worldwide, with rising incidence in younger populations. Red meat consumption has been proposed as a risk factor for CRC, though the evidence remains inconsistent. This systematic review and meta-analysis aimed to examine the associations between the consumption of beef, pork, and lamb with CRC, colon cancer (CC), and rectal cancer (RC) risk.

**Methods:**

The findings from 27 studies published between 1993 and 2024 were included, involving over 1 million participants from diverse geographical regions. Relative risks were calculated using random-effects meta-analysis, with subgroup and meta-regression analyses performed to assess potential sources of heterogeneity.

**Results:**

Beef consumption was significantly associated with increased CRC risk, with a 30% overall risk increase (95% *CI*: 1.10–1.54). The association with colon cancer (CC) was marginally significant (*RR* = 1.19, 95% *CI*: 0.99–1.43, *p* = 0.0585), while the link to rectal cancer (RC) was not statistically significant. Pork consumption was associated with a 17% increased CRC risk (95% *CI*: 1.09–1.25), with similar, nonsignificant trends for CC and RC. Lamb consumption was weakly associated with an 11% increase in CRC risk (95% *CI*: 1.02–1.21), though this was based on limited studies (*n* = 6), and no significant associations emerged for cancer subtypes. Study design and confounding factors influenced these associations, with case-control studies reporting stronger associations than cohort studies. Physical activity adjustments were pivotal, as studies without this adjustment consistently reported higher-risk estimates.

**Conclusion:**

These findings emphasise the importance of accounting several lifestyle factors in future research and public health guidance. While these results support current dietary guidelines recommending limited red meat consumption, they also underscore the complexity of diet-cancer relationships and the need for comprehensive, lifestyle-inclusive cancer prevention strategies.

**Supplementary Information:**

The online version contains supplementary material available at 10.1007/s12029-025-01247-3.

## Introduction

Cancer remains the second leading cause of death globally, affecting both developed and developing countries and imposing a significant societal burden [1]. According to Global Cancer Statistics (GLOBOCAN), nearly 20 million new cancer cases were diagnosed worldwide in 2022, with breast and lung cancers being the most prevalent [2]. Colorectal cancer (CRC) accounted for 10.7% of new cases and is the third most prevalent cancer. Notably, there is a trend toward earlier onset, especially among younger and middle-aged people between 20 and 54 years [3, 4], underscoring the urgent need to better understand and address associated risk factors. The increase in cancer incidence is attributed to various factors, including age, smoking, obesity, sedentary lifestyles, and the adoption of westernised diets [5, 6]. Among these factors, diets high in protein and fat but low in fibre may induce inflammation or new growth formation, potentially contributing to CRC development [7, 8].

Recently, research interest in the relationship between red meat intake and CRC risk has intensified. This complex relationship involves interactions between dietary components and host factors. The World Cancer Research Fund reports a convincing link between red and processed meat consumption and elevated CRC risk. Supporting this, epidemiological studies indicate a 17% increase in CRC risk for every 100 g of red meat consumed [9]. Consequently, dietary guidelines in several countries, including Australia, Norway, France, the UK, and the USA, recommend limiting red meat intake to no more than 450 g per week and minimising or avoiding processed meat.

The increased CRC risk linked to red meat consumption is attributed to specific meat components, such as *N*-nitroso compounds (NOC), heterocyclic aromatic amines (HCA), and polycyclic aromatic hydrocarbons (PAH), formed during processing or high-temperature cooking [10]. For example, NOC, potential carcinogens, result from the reaction of nitrites or nitrates with amines or amides during meat processing and can induce mutations like *KRAS*, commonly mutated in human tumours [11]. HCAs and PAHs, also classified as potential carcinogens by the International Agency for Research on Cancer (IARC), form during high-temperature cooking and are linked to CRC risk in animal studies and epidemiological research [12]. Heme iron, another important component found in red meat, contributes to NOC formation in the gastrointestinal tract. These compounds may contribute to CRC development through DNA adduct formation, oxidative stress, and dysregulation of proinflammatory cytokines [13, 14]. While much research has treated red meat as a uniform category, evidence suggests that different types of red meat may have varying associations with CRC risk [15, 16].

Our meta-analysis aims to collate findings on the associations between specific types of red meat consumption (beef, pork, and lamb) and the risk of colon cancer (CC) and rectal cancer (RC). Furthermore, we explore potential sources of heterogeneity in these relationships, including study design, publication year, geographical location, and adjustments for confounding factors such as physical activity and dietary fibre. By focusing exclusively on red meat (IARC Group 2 A) rather than processed meat (IARC Group 1), this study addresses a critical gap in current guidelines, which often conflate all red meat types despite variations in their carcinogenic potential. While the avoidance of processed meat is strongly recommended, our findings support moderating the intake of certain red meat types to refine existing precautionary advice. This targeted approach aims to enhance understanding of diet-cancer relationships, support more nuanced dietary recommendations, and inform future CRC prevention research.

## Materials and Methods

### Data Sources and Search Strategy

This systematic review and meta-analysis followed the Preferred Reporting Items for Systematic reviews and Meta-Analyses (PRISMA) guidelines [17]. A comprehensive literature search was performed across multiple databases, including Embase, Scopus, MEDLINE, and PubMed, up to August 2024, with results restricted to English-language publications. Search terms were designed to broadly capture relevant studies, combining keywords related to colorectal cancer (e.g. colorectal, colon, rectal, large bowel), cancer types (e.g. cancer, neoplasm, carcinoma), dietary exposures (e.g. meat, red meat, beef, pork, lamb, diet), study design (e.g. cohort, case–control, prospective), and risk measures (e.g. risk, rate, ratio, incidence). The identified studies were imported into Covidence for screening and selection.

### Study Selection and Data Extraction

Eligible studies were prospective investigations, including cohort, nested case–control, and case–control designs that reported relative risk (RR) estimates, such as hazard ratios, incidence rate ratios, or odds ratios, with 95% confidence intervals (CI). Studies needed to assess the relationship between specific types of red meat (beef, pork, lamb) and the risk of CRC, CC, or RC. Our inclusion criteria required studies to clearly distinguish between red meat types (beef, pork, lamb) and processed meat. Studies that did not separate red meat from processed meat or combined them in analyses were excluded to avoid confounding. Dietary assessment methods were categorised as frequency based (e.g. times/week) or portion based (e.g. g/day). We prioritised studies using validated tools like food frequency questionnaires (FFQs), regardless of whether the results were reported in frequency or portion-based terms. However, two studies using unvalidated FFQs were included due to their high Newcastle–Ottawa scale (NOS) scores (≥ 7) and unique focus on lamb consumption.

Data extraction was performed by one reviewer and verified by a second reviewer to ensure accuracy. Extracted data included study details (title, first author, publication year, country, study design, study population demographics, follow-up period) and dietary exposures (type of red meat, dietary assessment methods, portion size, and intake frequency). We also recorded outcome measures, including the total sample size, number of cancer cases, cancer site, adjusted RR with 95% CI, and confounding variables.

### Quality Assessment

The quality of included studies was evaluated using the NOS, which assesses three key domains: selection of study group, comparability of groups, and outcome ascertainment. For cohort studies, criteria included clearly defined cases, general population representation, and follow-up exceeding 10 years. Key confounders, such as age and sex, were also considered, alongside the reliability of red meat consumption and cancer outcome measures. For case–control studies, criteria included selecting cases and controls from the same population, ensuring cancer-free controls at baseline, adequately controlling for confounders, and ensuring valid exposure and outcome assessments. Two reviewers independently rated each study, resolving disagreements through consensus with a third reviewer. NOS scores ranged from 0 to 9 stars, with studies scoring 7 or above considered high quality.

### Statistical Analysis

The statistical analysis was conducted separately for each combination of red meat type (beef, pork, lamb) and cancer site (CRC, CC, RC), resulting in nine distinct analyses. For studies reporting separate data for males, females, or different races, we first conducted fixed-effect model meta-analyses to obtain an overall RR for each study. We then performed random-effects meta-analyses using the *metafor* package in R software for each meat type-cancer site combination. Between-study heterogeneity was quantified using the *I*^2^ statistic, with thresholds of < 25%, 25–75%, and > 75% indicating low, moderate, and high heterogeneity, respectively. Subgroup analyses were performed for combinations with substantial heterogeneity (*I*^2^ > 75%), exploring variability by factors such as study design, population characteristics, and adjustment for confounders. Meta-regression analyses evaluated potential sources of heterogeneity, incorporating variables such as body mass index (BMI), alcohol consumption, smoking, physical activity, and dietary fibre.

Publication bias was assessed for each meat type-cancer site combination using both visual (funnel plots) and quantitative methods (Egger’s regression test). For studies that reported meat consumption in portions (g/day), we conducted dose–response analyses for beef and pork in relation to CRC. Both linear and non-linear dose–response relationships were examined using random-effects meta-regression models. For non-linear associations, restricted cubic splines with three degrees of freedom were fitted. Model fit was compared using Akaike’s information criterion (AIC). For the linear model, relative risk per 50 g/day increase in meat (beef/pork) consumption is calculated. Results were visualised with bubble plots where bubble size represents case numbers in each study. Dose–response analysis for lamb was not conducted due to insufficient data. All statistical tests were two-sided, and a *p*-value < 0.05 was considered statistically significant. All analyses were performed using R software (version 4.4.0).

## Results

**Fig. 1:**
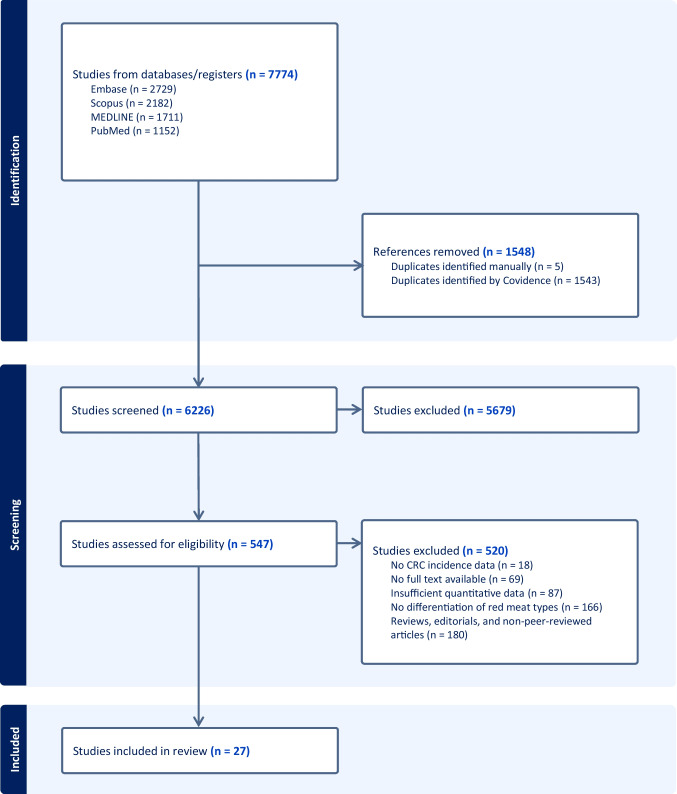
Flowchart of literature search and selection of studies

Initial literature search identified a total of 7774 studies. After removing 1548 duplicates (1543 identified by Covidence and 5 manually), 6226 studies remained for screening. The selection process is illustrated in Fig. 1. In the initial screening phase, 5679 studies were excluded based on their title and abstract review, leaving 547 studies for full-text evaluation. During the full-text review, 520 studies were further excluded. The reasons for exclusion were as follows: 180 were reviews, editorials, or non-peer-reviewed articles, 166 did not specify types of red meat, 87 lacked sufficient quantitative data, 69 had no available full texts, and 18 did not provide data on CRC incidence. Following this process, 27 studies met the eligibility criteria and were included in the systematic review and meta-analysis [16, 18–43].

The studies included were published between 1993 and 2024, representing over three decades of research (Supplementary Table 1). Of the included studies, 18 (66.7%) were case–control studies, and 33.3% were cohort studies. Cohort studies had follow-up periods ranging from 4.8 to 26 years, with a median follow-up of 10.6 years. These studies covered a wide geographical range with two from Africa (Morocco and Uganda), five from Asia (China, India, Israel, Japan, and Malaysia), eight from Europe (Denmark, France, Germany, the Netherlands, and Sweden), eight from North America (Canada, Mexico, and USA), and four from South America (Argentina, Brazil, and Uruguay). This diversity provides a global perspective on the relationship between red meat consumption and CRC risk across varied populations and dietary patterns.

The total number of participants across these studies was 1,028,166, with individual sample sizes ranging from 270 to 478,040 participants. The largest study, conducted by Norat et al. (2005), involved multiple European countries [26]. The age range of participants was broad, with most studies focusing on adults aged 40 and above, though some included younger participants, resulting in an overall range from 18 to 89 years. Gender distribution was reported in most studies, with many achieving relatively balanced representation.

### Beef Consumption and CRC

**Fig. 2:**
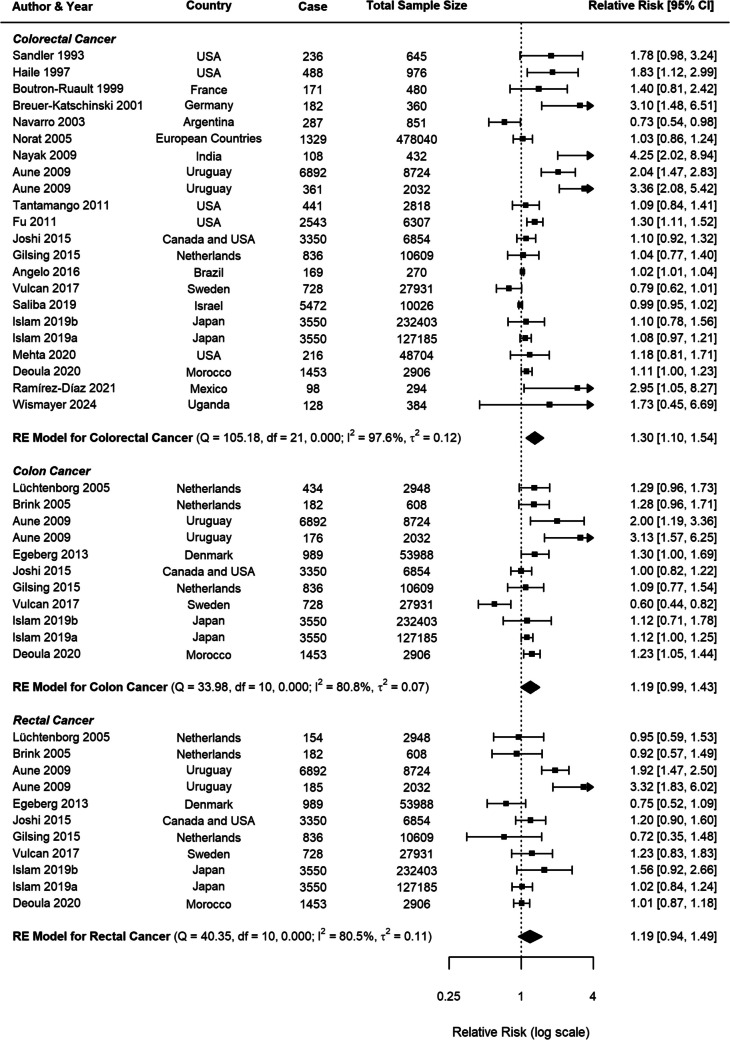
Forest plot of relative risk of colorectal, colon, and rectal cancer with high versus low beef consumption

For beef consumption, the meta-analysis comprised 22 studies for CRC and 11 studies specifically examining CC and RC (Fig. 2). A positive association was identified between beef consumption and CRC risk, with a pooled RR of 1.30 (95% *CI*: 1.10–1.54, *p* = 0.0022). When analysing specific cancer subtypes, a possible relationship was observed for CC with an RR of 1.19 (95% *CI*: 0.99–1.43, *p* = 0.0585), while RC demonstrated a nonsignificant positive association (*RR* = 1.19, 95% *CI*: 0.95–1.49, *p* = 0.1408).

To evaluate potential publication bias and small-study effects, funnel plots were created for overall CRC, as well as for CC and RC separately (Supplementary Figs. 1, 2, 3). These funnel plots, combined with Egger’s test results, suggest that while publication bias may not be a major concern for CC and RC individually, it could be influencing the results for overall CRC (*p* < 0.0001).

**Fig. 3:**
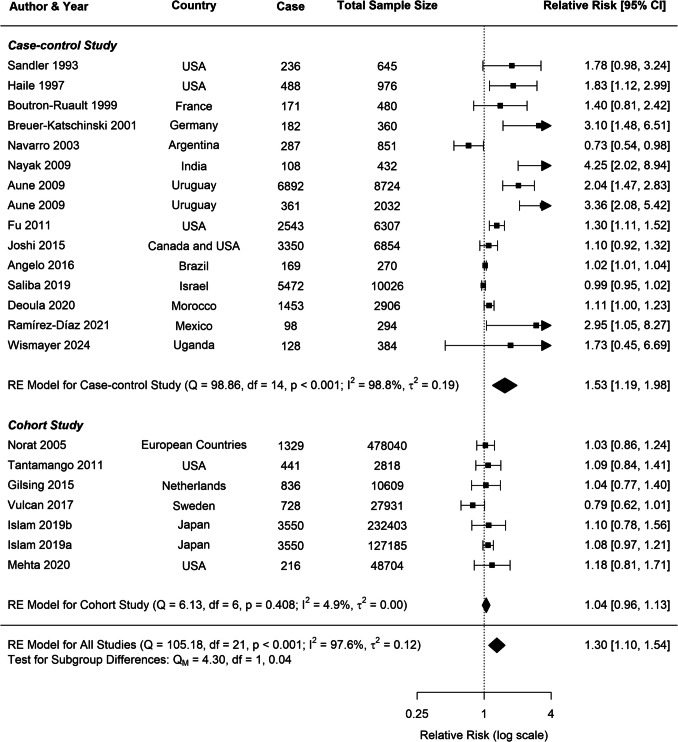
Forest plot of cohort and case–control studies of colorectal cancer risk associated with beef consumption

**Fig. 4:**
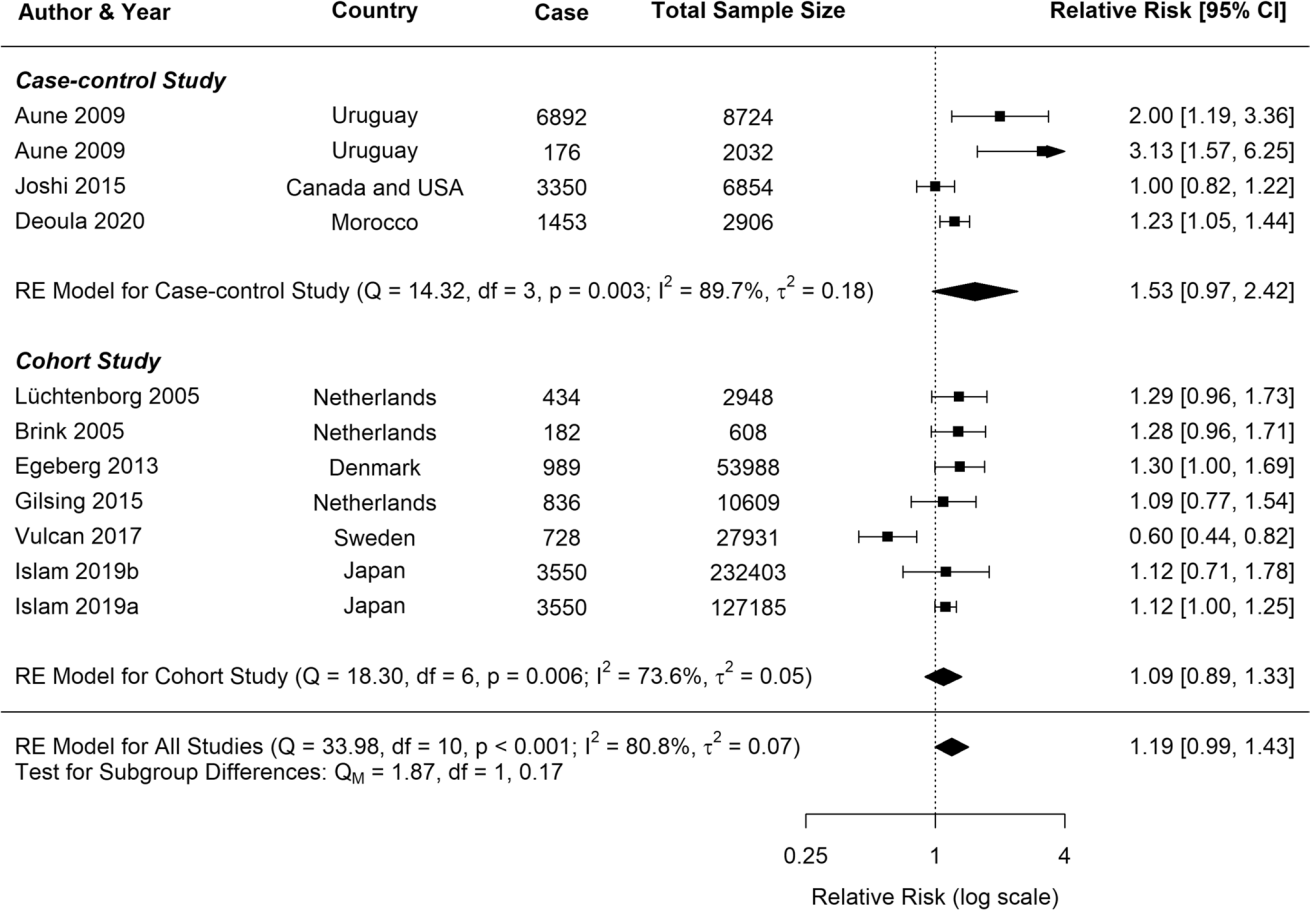
Forest plot of cohort and case–control studies of colon cancer risk associated with beef consumption

**Fig. 5:**
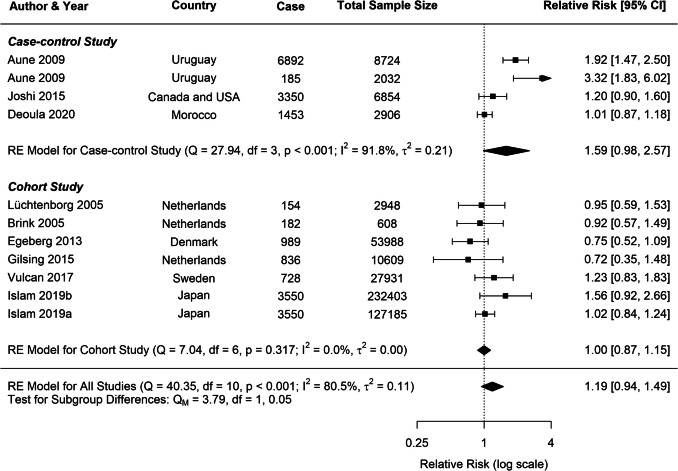
Forest plot of cohort and case–control studies of rectal cancer risk associated with beef consumption

Substantial heterogeneity was observed across all analyses for beef consumption, with *I*^2^ values of 97.64% for CRC, 80.85% for CC, and 80.49% for RC, indicating considerable variability in the reported associations. To explore potential sources of this heterogeneity, several subgroup analyses and meta-regressions were conducted. Our subgroup analyses revealed several important findings (Table 1). Consistently across CRC, CC, and RC, stronger associations were observed in case–control studies compared to cohort studies (Figs. 3, 4, 5). For CRC, case–control studies reported an RR of 1.53 (95% *CI*: 1.19–1.98), whereas cohort studies showed an RR of 1.04 (95% *CI*: 0.96–1.13). This difference was statistically significant (*p* = 0.0382). A similar pattern was observed for RC (*p* = 0.0516), suggesting that the study design is a significant factor contributing to heterogeneity in the results.

**Table 1:** Stratified meta-analyses of beef consumption and colorectal, colon, and rectal cancer risk

Subgroups	Colorectal cancer				Colon cancer				Rectal cancer			
	*N*	RR (95% *CI*)	*P*	*I*^2^ (%)	*N*	RR (95% *CI*)	*P*	*I*^2^ (%)	*N*	RR (95% *CI*)	*P*	*I*^2^ (%)
All studies	22	1.30 (1.10–1.54)	**0.0022**	97.64	11	1.19 (0.99–1.43)	0.0585	80.85	11	1.19 (0.95–1.49)	0.1408	80.49
Study type				97.28				80.60				73.72
*Case control*	15	1.53 (1.19–1.98)	**0.00**	98.82	4	1.53 (0.97–2.42)	0.07	89.69	4	1.59 (0.98–2.57)	0.06	91.83
*Cohort*	7	1.04 (0.96–1.13)	0.32	4.93	7	1.09 (0.89–1.33)	0.40	73.63	7	1.00 (0.87–1.15)	0.98	0.00
Publication year				96.82				70.72				81.47
*After 2015*	11	1.03 (0.99–1.08)	0.16	44.19	6	1.01 (0.83–1.23)	0.89	78.88	6	1.06 (0.96–1.18)	0.27	0.04
*Before 2015*	11	1.63 (1.20–2.23)	**0.00**	89.54	5	1.40 (1.20–1.63)	**0.00**	0.00	5	1.31 (0.78–2.22)	0.31	87.61
Continent				92.80				68.71				33.10
*Africa*	2	1.11 (1.00–1.23)	**0.04**	0.00	1	1.23 (1.05–1.44)	-	-	1	1.01 (0.86–1.17)	-	-
*Asia*	4	1.37 (0.78–2.40)	0.27	98.48	2	1.12 (1.00–1.25)	0.05	0.00	2	1.17 (0.79–1.73)	0.43	53.36
*Europe*	5	1.17 (0.82–1.66)	0.39	83.05	5	1.07 (0.80–1.44)	0.63	78.53	5	0.92 (0.74–1.14)	0.46	11.34
*North America*	7	1.24 (1.11–1.40)	**0.00**	14.74	1	1.0 (0.8–1.2)	-	-	1	1.2 (0.9–1.6)	-	-
*South America*	4	1.47 (0.76–2.84)	0.25	96.35	2	2.35 (1.54–3.59)	**0.00**	3.19	2	2.36 (1.40–3.97)	**0.00**	63.19
Sample size				94.07				84.21				82.83
> *1500*	13	1.18 (1.01–1.38)	**0.04**	92.45	10	1.19 (0.97–1.46)	0.10	84.21	10	1.21 (0.95–1.55)	0.12	82.83
*500*–*1500*	3	1.28 (0.68–2.41)	0.44	82.45	1	1.28 (0.96–1.72)	-	-	1	0.92 (0.57–1.49)	-	-
< *500*	6	1.99 (1.19–3.33)	**0.01**	78.02	0	-	-	-	0	-	-	-
Measure methods				94.43				82.02				62.82
*Frequency*	9	1.51 (1.12–2.02)	**0.01**	90.72	2	1.48 (0.84–2.61)	0.17	62.49	2	1.84 (1.46–2.33)	**0.00**	0.00
*Portion*	13	1.20 (0.98–1.47)	0.15	95.46	9	1.15 (0.95–1.39)	0.15	82.51	9	1.08 (0.87–1.33)	0.48	71.87
BMI				94.01				84.05				79.85
*Yes*	15	1.13 (1.03–1.24)	**0.01**	66.20	6	1.13 (1.05–1.23)	**0.00**	0.00	6	1.04 (0.94–1.16)	0.43	0.01
*No*	7	1.57 (1.00–2.46)	**0.05**	96.45	5	1.37 (0.82–2.30)	0.23	89.85	5	1.38 (0.84–2.27)	0.21	87.31
Alcohol consumption				97.87				83.58				82.02
*Yes*	14	1.30 (1.05–1.62)	**0.02**	98.62	8	1.23 (0.93–1.63)	0.15	88.75	8	1.26 (0.91–1.73)	0.16	87.66
*No*	8	1.31 (0.98–1.76)	0.07	78.48	3	1.15 (0.96–1.38)	0.14	34.53	3	1.08 (0.87–1.35)	0.49	0.00
Smoking				97.87				82.63				82.94
*Yes*	14	1.31 (1.05–1.63)	**0.02**	98.65	10	1.22 (1.00–1.50)	0.06	82.63	10	1.19 (0.92–1.54)	0.20	82.94
*No*	8	1.29 (0.97–1.73)	0.08	77.02	1	1.0 (0.8–1.2)	-	-	1	1.2 (0.9–1.6)	-	-
Physical activity				90.35				74.37				74.18
*Yes*	12	1.06 (0.97–1.15)	0.20	69.59	7	1.05 (0.88–1.26)	0.58	76.85	7	1.03 (0.93–1.14)	0.52	0.00
*No*	10	1.91 (1.36–2.67)	**0.00**	87.16	4	1.62 (1.14–2.31)	**0.01**	66.18	4	1.52 (0.86–2.70)	0.15	85.59
Dietary fibre				94.47				81.46				81.60
*Yes*	10	1.26 (0.97–1.63)	0.09	96.70	7	1.24 (1.06–1.46)	**0.01**	62.89	7	1.32 (0.95–1.83)	0.10	89.61
*No*	12	1.35 (1.07–1.71)	**0.01**	89.37	4	1.02 (0.72–1.46)	0.91	81.10	4	1.01 (0.79–1.28)	0.96	0.00

N number of studies included in the analysis, RR relative risk, CI confidence interval, P p-value, I^2^ I-squared statistic

Publication year emerged as another significant moderator influencing the observed associations. Studies published before 2015 reported stronger associations for both CRC and CC compared to those published after 2015. For CRC, studies published before 2015 had an RR of 1.63 (95% *CI*: 1.20–2.23), whereas post-2015 studies showed a weaker association (*RR*: 1.03, 95% *CI*: 0.99–1.08), with this difference being statistically significant (*p* = 0.0178). A similar trend was evident for CC (*p* = 0.0145).

Geographical differences were particularly pronounced for RC, with South American studies reporting the strongest association (*RR* = 2.36, 95% *CI*: 1.40–3.97), followed by Asian studies (*RR* = 1.17, 95% *CI*: 0.79–1.73) and European studies (*RR* = 0.92, 95% *CI*: 0.74–1.14). These differences were statistically significant (*p* = 0.0018), underscoring the role of regional dietary patterns and other contextual factors.

For all cancer groups (CRC, CC, and RC), studies which were not adjusted for physical activity reported substantially stronger associations between beef consumption and cancer risk. For CRC, non-adjusted studies yielded an RR of 1.91 (95% *CI*: 1.36–2.67), compared to an RR of 1.06 (95% *CI*: 0.97–1.15) in adjusted studies. This difference was highly significant (*p* = 0.0007), with similar patterns observed for CC (*p* = 0.0219) and RC (*p* = 0.0888).

Meta-regression analysis for CRC further substantiated these findings. The model explained 26.78% of the heterogeneity in the beef-CRC association, with physical activity emerging as the most significant moderator (*p* = 0.0006). Other potential moderators, including BMI, alcohol consumption, smoking, and dietary fibre, did not significantly impact the relationship. For CC, the meta-regression model explained a larger proportion of heterogeneity (*R*^2^ = 52.76%), identifying dietary fibre as an important factor (*p* = 0.0190). Conversely, no clear influential factors were identified for RC, potentially due to the smaller number of studies (*n* = 6).

**Fig. 6:**
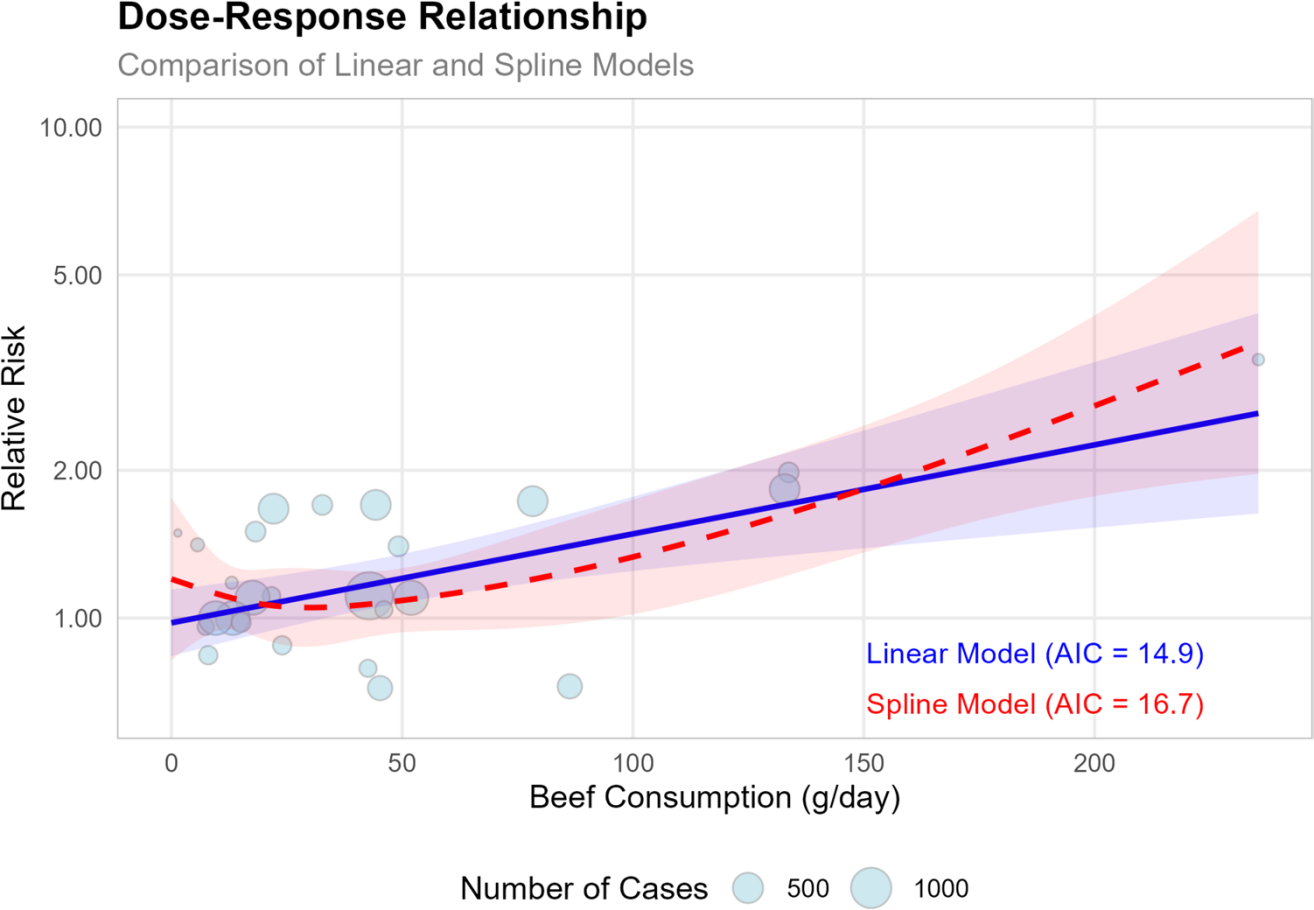
The plot compares linear and spline models, with the size of each bubble representing the number of cases in each study

To further explore the beef-CRC relationship, a dose–response analysis was conducted (Fig. 6). The results showed that each 50 g/day increase in beef consumption was associated with a 23% higher risk (*RR* = 1.23, 95% *CI*: 1.09–1.39, *p* = 0.0006). The spline model also confirmed the positive association, highlighting a particularly strong relationship at higher levels of beef consumption (*p* = 0.0002), further supporting a dose-dependent relationship between beef intake and CRC risk.

### Pork Consumption and CRC

**Fig. 7:**
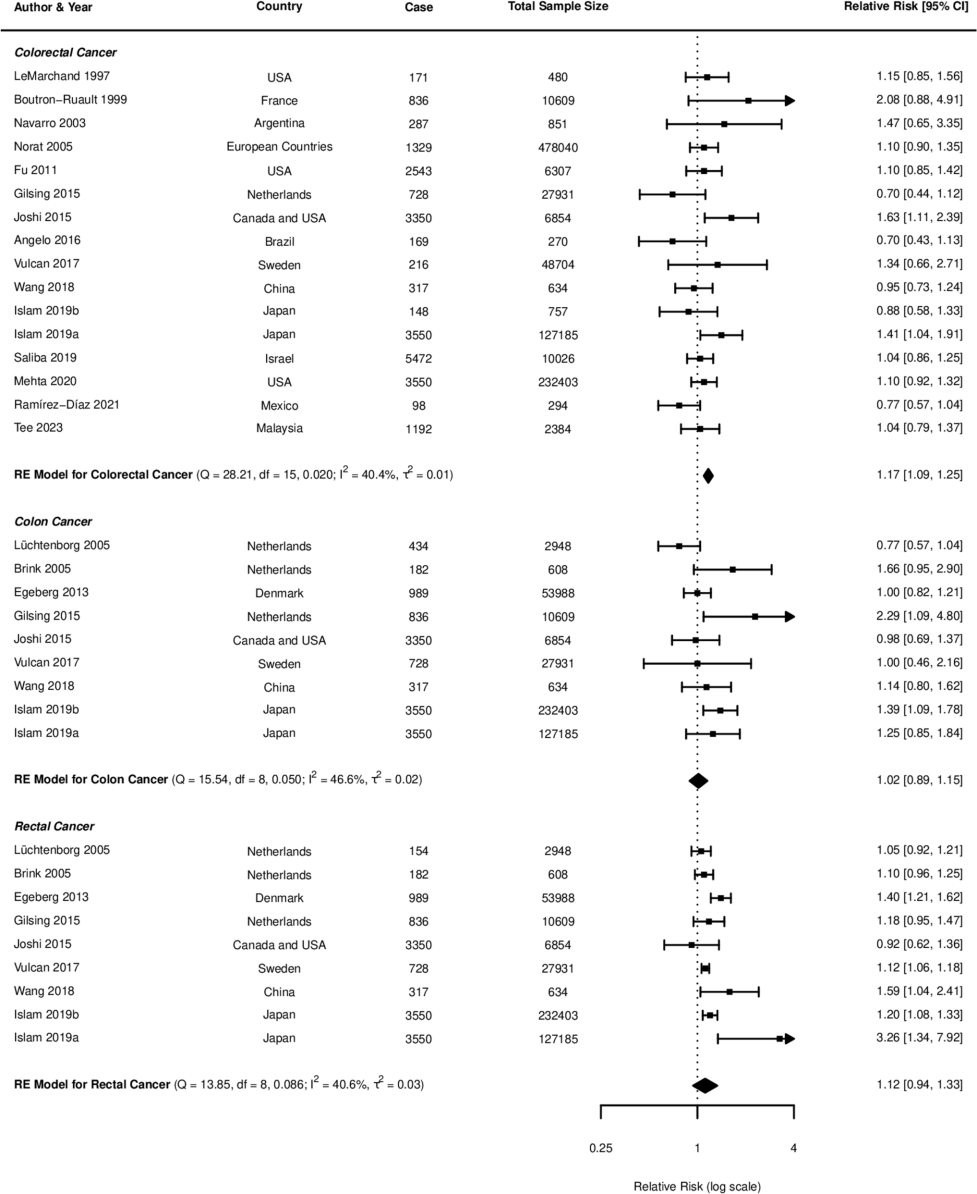
Forest plot of relative risk of colorectal, colon, and rectal cancer with high versus low pork consumption

The current study identified significant associations between pork consumption and various cancer risks (Fig. 7). For CRC, a random-effects meta-analysis of 16 studies showed a significant positive association, with an RR of 1.17 (95% *CI*: 1.09–1.25, *p* < 0.0001). However, for CC, the analysis of nine studies did not reveal a clear association (*RR* = 1.02, 95% *CI*: 0.89–1.15, *p* = 0.8172). Similarly, the pooled analysis for RC showed a nonsignificant positive trend, with an RR of 1.12 (95% *CI*: 0.94–1.33, *p* = 0.2113).

To assess potential publication bias in our meta-analysis, we generated funnel plots for CRC, CC, and RC (Supplementary Figs. 4, 5, and 6). Across all three funnel plots, studies with larger sample sizes (and lower standard errors) were observed to cluster closely around the overall effect estimate. In contrast, studies with smaller sample sizes showed greater variability in their reported effect sizes.

Despite these observations, Egger’s test indicated no significant asymmetry across any of the cancer types (*CRC*: *p* = 0.111; *CC*: *p* = 0.6023; *RC*: *p* = 0.4786). These results suggest minimal publication bias in the included studies, providing greater confidence in the overall findings of this analysis.

**Fig. 8:**
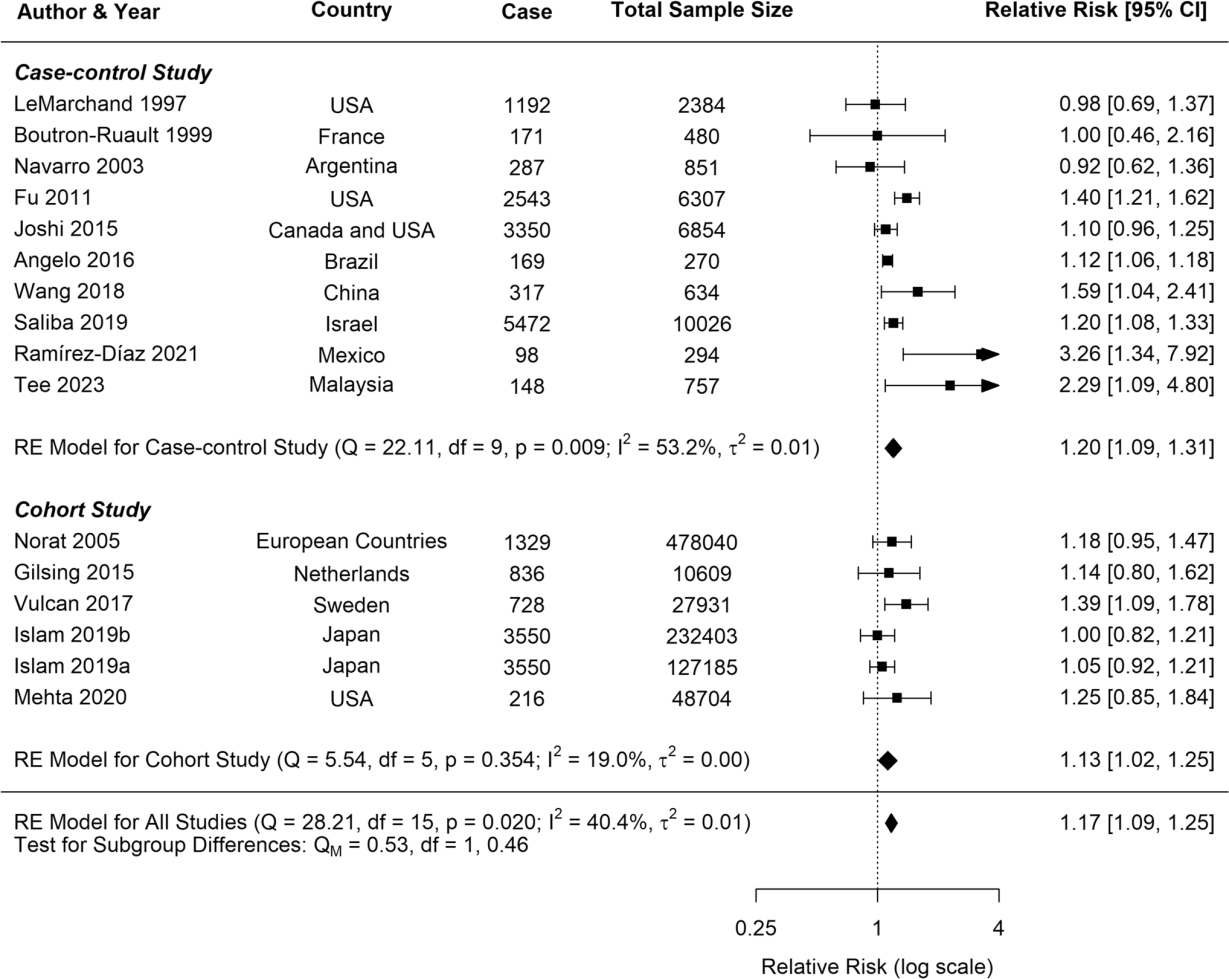
Forest plot of cohort and case–control studies of colorectal cancer risk associated with pork consumption

**Fig. 9:**
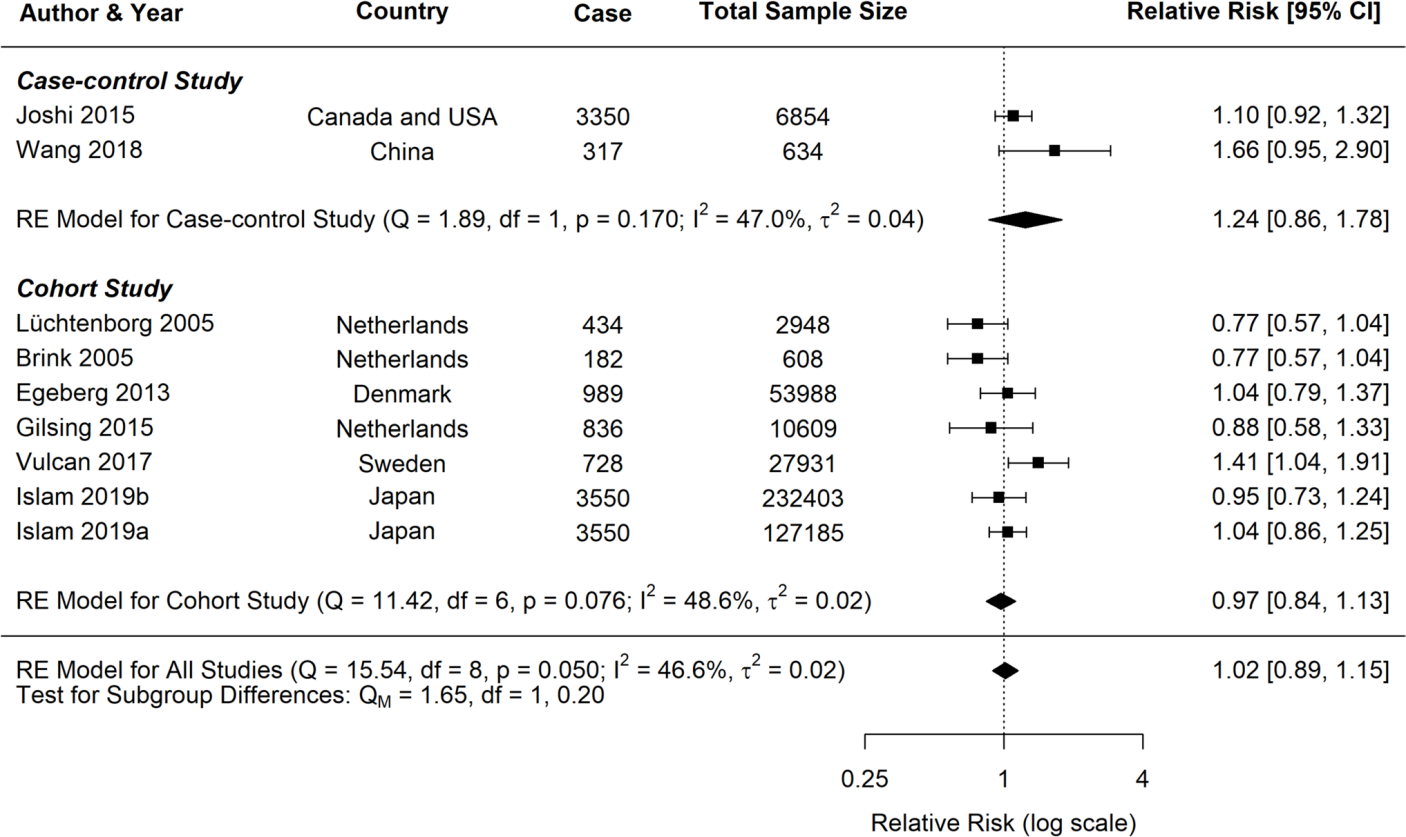
Forest plot of cohort and case–control studies of colon cancer risk associated with pork consumption

**Fig. 10:**
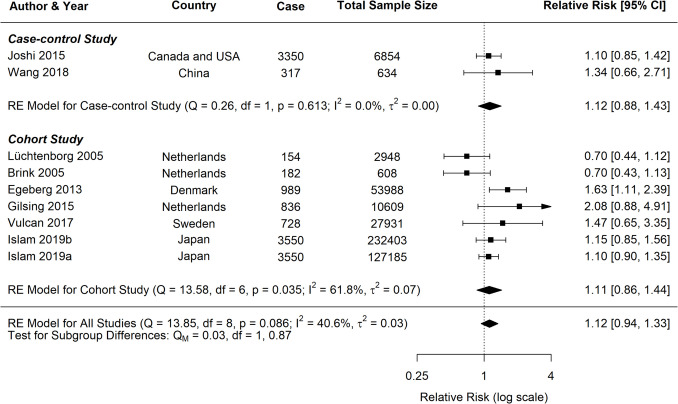
Forest plot of cohort and case–control studies of rectal cancer risk associated with pork consumption

A moderate heterogeneity was observed across studies for all cancer types (*CRC*: *I*^2^ = 40.42%; *CC*: *I*^2^ = 46.59%; *RC*: *I*^2^ = 40.62%), which is common in dietary meta-analysis due to variations in study designs and populations. Subgroup analyses for CRC consistently indicated positive associations across various study characteristics (Table 2). Both case–control (*RR* = 1.20, 95% *CI*: 1.09–1.31) and cohort studies (*RR* = 1.13, 95% *CI*: 1.02–1.25) demonstrated significant positive associations with CRC risk (Fig. 8). In contrast, analyses for CC and RC revealed no clear associations, though a trend toward increased risk was observed for both study types (Figs. 9, 10).

**Table 2:** Stratified meta-analyses of pork consumption and colorectal, colon, and rectal cancer risk

Subgroups	Colorectal cancer				Colon cancer				Rectal cancer			
	*N*	RR (95% *CI*)	*P*	*I*^2^ (%)	*N*	RR (95% *CI*)	*P*	*I*^2^ (%)	*N*	RR (95% *CI*)	*P*	*I*^2^ (%)
All studies	16	1.17 (1.09–1.25)	** < 0.0001**	40.42	9	1.02 (0.89–1.15)	0.8172	46.59	9	1.12 (0.94–1.33)	0.2113	40.62
Study type				42.25				48.42				53.21
*Case–control study*	10	1.20 (1.09–1.31)	**0.00**	53.24	2	1.24 (0.86–1.78)	0.25	46.98	2	1.12 (0.88–1.43)	0.34	0.00
*Cohort study*	6	1.13 (1.02–1.25)	**0.02**	19.00	7	0.97 (0.84–1.13)	0.70	48.59	7	1.11 (0.86–1.44)	0.41	61.83
Publication year				32.22				13.45				54.53
*After 2015*	11	1.14 (1.09–1.18)	**0.00**	0.07	6	1.09 (0.98–1.21)	0.11	0.00	6	1.14 (1.00–1.31)	**0.05**	0.00
*Before 2015*	5	1.16 (0.96–1.39)	**0.12**	49.73	3	0.86 (0.70–1.05)	0.14	31.7	3	0.94 (0.53–1.66)	0.84	79.53
Continent				40.61				58.56				61.67
*Africa*	0	-	**-**	-	0	-	-	-	0	-	-	-
*Asia*	5	1.15 (1.02–1.30)	**0.03**	44.96	3	1.05 (0.90–1.21)	0.55	0.01	3	1.13 (0.96–1.33)	0.16	0.00
*Europe*	4	1.24 (1.07–1.43)	**0.00**	0.00	5	0.95 (0.76–1.20)	0.68	63.02	5	1.13 (0.72–1.77)	0.59	69.1
*North America*	5	1.23 (1.04–1.47)	**0.02**	58.25	1	1.1 (0.9–1.3)	-	-	1	1.1 (0.9–1.5)	-	-
*South America*	2	1.12 (1.06–1.18)	**0.00**	0.00	0	-	-	-	0	-	-	-
Sample size				43.75				52.21				33.02
> *1500*	10	1.17 (1.08–1.26)	**0.00**	43.83	7	1.03 (0.92–1.15)	0.61	25.49	7	1.14 (1.00–1.31)	**0.05**	6.72
*500–1500*	3	1.40 (0.85–2.32)	0.18	67.57	2	1.09 (0.51–2.31)	0.82	82.27	2	0.92 (0.49–1.71)	0.78	54.20
< *500*	3	1.40 (0.76–2.59)	0.28	69.40	0	-	-	-	0	-	-	-
Measure methods				22.29				57.44				53.23
*Frequency*	6	1.36 (1.10–1.70)	**0.01**	78.98	2	1.19 (0.70–2.03)	0.53	67.83	2	1.18 (0.89–1.56)	0.26	0.00
*Portion*	10	1.12 (1.07–1.17)	**0.00**	0.00	7	1.00 (0.86–1.16)	0.97	53.73	7	1.10 (0.86–1.42)	0.44	62.79
BMI				42.58				55.5				52.17
*Yes*	11	1.16 (1.06–1.27)	**0.00**	46.41	6	1.01 (0.90–1.13)	0.86	10.47	6	1.09 (0.96–1.25)	0.18	0.01
*No*	5	1.20 (1.06–1.35)	**0.00**	30.98	3	1.04 (0.74–1.46)	0.81	74.98	3	1.17 (0.66–2.07)	0.58	70.53
Alcohol consumption				44.79				46.84				35.46
*Yes*	10	1.17 (1.09–1.27)	**0.00**	53.17	6	1.08 (0.95–1.23)	0.23	13.21	6	1.26 (1.05–1.52)	**0.01**	19.93
*No*	6	1.25 (0.96–1.62)	0.10	52.42	3	0.89 (0.69–1.15)	0.36	65.4	3	0.86 (0.61–1.20)	0.37	53.75
Smoking				48.00				48.42				53.21
*Yes*	9	1.16 (1.08–1.26)	**0.00**	53.78	7	0.97 (0.84–1.13)	0.7	48.59	7	1.11 (0.86–1.44)	0.41	61.83
*No*	7	1.29 (1.02–1.64)	**0.03**	53.02	2	1.24 (0.86–1.78)	0.25	46.98	2	1.12 (0.88–1.43)	0.34	0.00
Physical activity				39.9				0.02				0.00
*Yes*	13	1.18 (1.09–1.27)	**0.00**	41.08	7	1.08 (0.98–1.19)	0.11	0.00	7	1.19 (1.05–1.35)	**0.01**	0.00
*No*	3	1.40 (0.76–2.59)	0.28	69.40	2	0.77 (0.62–0.95)	**0.02**	0.00	2	0.70 (0.50–0.98)	0.04	0.00
Dietary fibre				36.57				49.28				0.00
*Yes*	9	1.12 (1.05–1.20)	**0.00**	7.45	5	1.06 (0.96–1.18)	0.26	0.04	5	1.17 (1.03–1.33)	**0.02**	0.00
*No*	7	1.26 (1.11–1.43)	**0.00**	49.03	4	0.93 (0.69–1.25)	0.62	69.82	4	1.00 (0.60–1.65)	0.99	61.46

N number of studies included in the analysis, RR relative risk, CI confidence interval, P p-value, I^2^ I-squared statistic

Studies published after 2015 demonstrated more consistent positive associations across all cancer groups, particularly for CRC (*RR* = 1.14, 95% *CI*: 1.09–1.18, *p* < 0.0001) and marginally for RC (*RR* = 1.14, 95% *CI*: 1.00–1.31, *p* = 0.05), as outlined in Table 2. More extensive studies (> 1500 participants) showed a significant association for both CRC (*RR* = 1.17, 95% *CI*: 1.08–1.26, *p* < 0.0001) and RC (*RR* = 1.14, 95% *CI*: 1.00–1.31, *p* = 0.05).

Analysis of measurement methodologies indicated significant positive associations for CRC using both frequency-based (*RR* = 1.36, 95% *CI*: 1.10–1.70) and portion-based approaches (*RR* = 1.12, 95% *CI*: 1.07–1.17). The influence of confounding factors varied by cancer type. In CRC studies, the association remained significant after adjustments for various confounding factors such as BMI, alcohol consumption, smoking, physical activity, and dietary fibre intake. For RC, studies that adjusted for physical activity reported a positive association (*RR* = 1.19, 95% *CI*: 1.05–1.35, *p* = 0.01), while those adjusted for dietary fibre also indicated a similar association (*RR* = 1.17, 95% *CI*: 1.03–1.33, *p* = 0.02).

Meta-regression analyses were performed for CRC, CC, and RC. For CRC, the mixed-effects model reduced residual heterogeneity to 15.46%, accounting for 52.76% of the observed heterogeneity (*R*^2^). In contrast, both CC and RC models demonstrated complete heterogeneity resolution, with *I*^2^ = 0% and *R*^2^ = 100%, suggesting that the included moderators fully explained the variation in effect sizes for these cancer types. Adjustment for dietary fibre emerged as a significant moderator for CRC (*p* = 0.0190), suggesting that studies accounting for dietary fibre reported stronger associations between pork consumption and CRC risk. However, this effect was not significant for CC (*p* = 0.4276) or RC (*p* = 0.3467). Other individual moderators did not show statistically significant effects, potentially due to the limited number of studies and reduced statistical power.

**Fig. 11:**
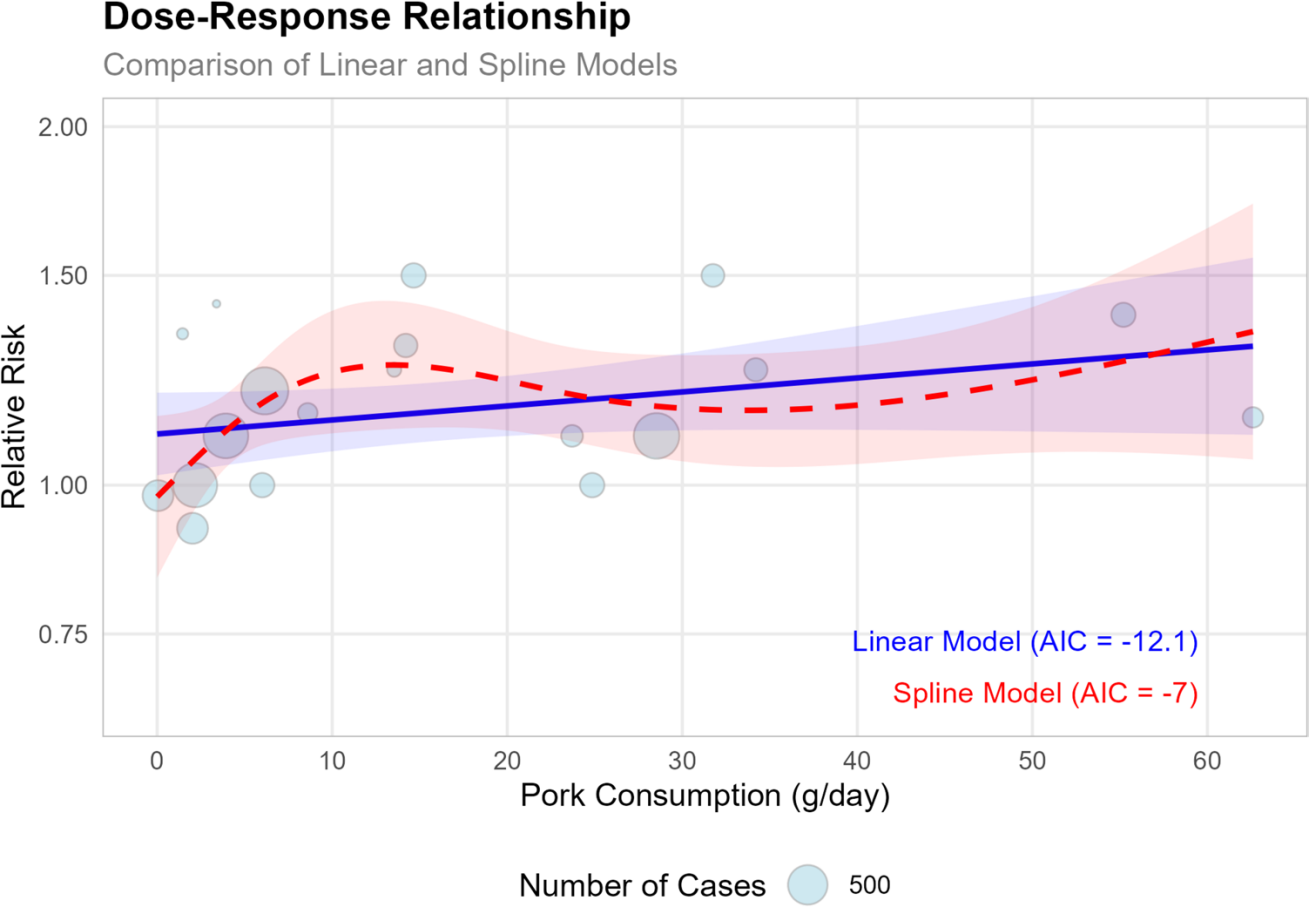
The plot compares linear and spline models, with the size of each bubble representing the number of cases in each study

A dose–response analysis was also conducted (Fig. 11), which showed each 50 g/day increase in pork consumption was associated with a 15% higher risk of CRC (*RR* = 1.15, 95% *CI*: 0.96–1.36, *p* = 0.127). The linear model (*AIC* = − 12.1) provided a better fit than the spline model (*AIC* = − 7). These dose–response findings support our overall meta-analysis results (*RR* = 1.17, 95% *CI*: 1.09 − 1.25), showing a significant positive association between pork consumption and CRC risk.

### Lamb Consumption and CRC

**Fig. 12:**
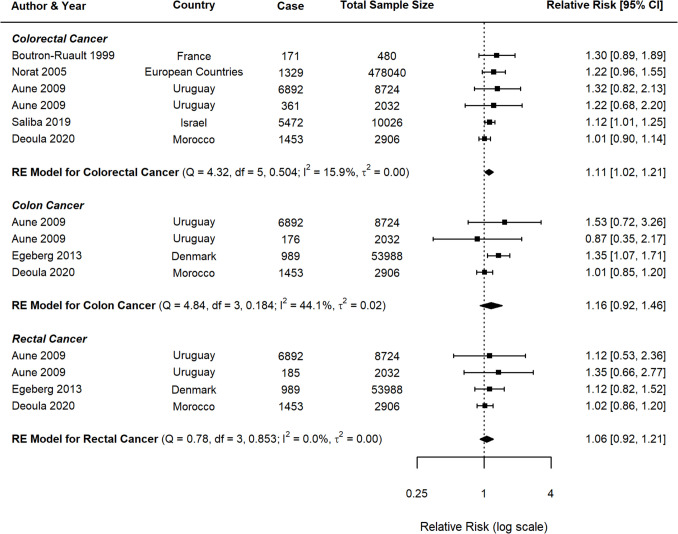
Forest plot of relative risk of colorectal, colon, and rectal cancer with high versus low lamb consumption

For lamb consumption, the meta-analysis included six studies for CRC, four for CC, and four for RC. The results are summarised in the forest plots (Fig. 12), which display the RR and CI for each study, as well as the pooled estimates. For CRC, the random-effects model indicated a significant association, with a pooled RR of 1.11 (95% *CI*: 1.02–1.21, *p* = 0.0216) and low heterogeneity (*I*^2^ = 15.9%). For CC, the analysis revealed a positive but nonsignificant association (*RR* = 1.16, 95% *CI*: 0.92–1.46, *p* = 0.184), with moderate heterogeneity (*I*^2^ = 44.1%). On the other hand, for RC, no clear association was found (*RR* = 1.06, 95% *CI*: 0.92–1.21, *p* = 0.853), with negligible heterogeneity (*I*^2^ = 0.0%).

Of the three red meat types, lamb had the fewest number of studies, with only one prospective cohort study available. While a modest association was observed for CRC (*p* = 0.0216), the limited data preclude reliable conclusions. Comparisons between study designs (cohort vs. case control) were limited by the scarcity of cohort data (one study) across all cancer types (CRC, CC, and RC). These results are presented in Supplementary Figs. 7, 8, and 9 for transparency but should be interpreted cautiously. To evaluate publication bias and small-study effects, funnel plots (Supplementary Figs. 10, 11, and 12) and Egger’s regression test were performed for each cancer subgroup (CRC, CC, and RC). The results suggested that publication bias is unlikely to significantly influence the findings, as Egger’s tests did not detect significant funnel plot asymmetry for any of the cancer types.

Subgroup analyses were performed to examine the influence of publication year, continent, BMI adjustment, and other lifestyle factors (Table 3). For CRC, studies published before 2015 (*n* = 4) reported a stronger association with lamb consumption (*RR* = 1.25, 95% *CI*: 1.05–1.49) compared to those published after 2015 (*n* = 2; *RR* = 1.07, 95% *CI*: 0.96–1.19), though the latter estimate is limited by the small sample size. A similar trend was observed for CC, with pre-2015 studies (*n* = 3) demonstrating a significant association (*RR* = 1.33, 95% *CI*: 1.07–1.65). The correlation between lamb intake and CRC risk was more pronounced in studies that did not adjust for BMI (*RR* = 1.25, 95% *CI*: 1.05–1.49) compared to those that accounted for BMI (*RR* = 1.07, 95% *CI*: 0.96–1.19). Meta-regression analyses further suggested that BMI adjustment significantly influenced outcomes for CC (*p* = 0.0487); however, this finding is not robust due to the small number of studies (*n* = 4). For CRC and RC, no consistent modifying factors were identified.

**Table 3:** Stratified meta-analyses of lamb consumption and colorectal, colon, and rectal cancer risk

Subgroups	Colorectal cancer				Colon cancer				Rectal cancer			
	*N*	RR (95% *CI*)	*P*	*I*^2^ (%)	*N*	RR (95% *CI*)	*P*	*I*^2^ (%)	*N*	RR (95% *CI*)	*P*	*I*^2^ (%)
All studies	6	1.11 (1.02–1.21)	**0.0200**	15.91	4	1.16 (0.92–1.46)	0.2123	44.09	4	1.06 (0.92–1.21)	0.4541	0.00
Study type				15.64				0.00				0.00
*Case–control study*	5	1.09 (1.00–1.20)	0.06	15.64	3	1.02 (0.87–1.20)	0.77	0.00	3	1.04 (0.89–1.22)	0.64	0.00
*Cohort study*	1	1.22 (0.96–1.55)	-	-	1	1.35 (1.07–1.71)	-	-	1	1.12 (0.83–1.53)	-	-
Publication year				15.64				0.00				0.00
*After 2015*	2	1.07 (0.96–1.19)	0.21	42.87	1	1.01 (0.85–1.19)	-	-	1	1.01 (0.85–1.19)	-	-
*Before 2015*	4	1.25 (1.05–1.49)	**0.01**	0.00	3	1.33 (1.07–1.65)	**0.01**	0.00	3	1.15 (0.88–1.49)	0.30	0.00
Continent				0.00				0.00				0.00
*Africa*	1	1.01 (0.90–1.14)	-	-	1	1.01 (0.85–1.19)	-	-	1	1.02 (0.86–1.20)	-	-
*Asia*	1	1.12 (1.01–1.25)	-	-	0	-	-	-	0	-	-	-
*Europe*	2	1.24 (1.02–1.52)	**0.03**	0.00	1	1.35 (1.07–1.71)	-	-	1	1.12 (0.83–1.53)		
*North America*	0	-	-	-	0	-	-	-	0	-	-	-
*South America*	2	1.28 (0.88–1.85)	0.19	0.00	2	1.22 (0.68–2.18)	0.51	0.00	2	1.23 (0.74–2.07)	0.43	0.00
Sample size				15.92			-	-			-	-
> *1500*	5	1.10 (1.01–1.20)	**0.04**	15.92	4	1.16 (0.92–1.46)	0.21	44.09	4	1.06 (0.92–1.21)	0.45	0.00
*500–1500*	0	-	-	-	0	-	-	-	0	-	-	-
< *500*	1	1.36 (0.85–2.18)	-	-	0	-	-	-	0	-	-	-
Measure methods				22.53				55.36				0.00
*Frequency*	2	1.13 (1.02–1.25)	**0.02**	0.00	1	1.53 (0.72–3.26)	-	-	1	1.12 (0.53–2.35)	-	-
*Portion*	4	1.11 (0.96–1.30)	0.17	30.17	3	1.13 (0.88–1.45)	0.34	55.36	3	1.05 (0.91–1.22)	0.48	0.00
BMI				9.76				0.00				0.00
*Yes*	2	1.07 (0.96–1.19)	0.21	42.87	1	1.01 (0.85–1.19)	-	-	1	1.02 (0.86–1.20)	-	-
*No*	4	1.25 (1.05–1.49)	**0.01**	0.00	3	1.33 (1.07–1.65)	**0.01**	29.49	3	1.15 (0.88–1.49)	0.30	0.00
Alcohol consumption				15.92			-	-			-	-
*Yes*	5	1.10 (1.01–1.20)	**0.04**	15.92	4	1.16 (0.92–1.46)	0.21	44.09	4	1.06 (0.92–1.21)	0.45	0.00
*No*	1	1.3 (0.9–1.9)	-	-	0	-	-	-	0	-	-	-
Smoking				15.92			-	-			-	-
*Yes*	5	1.10 (1.01–1.20)	**0.04**	15.92	4	1.16 (0.92–1.46)	0.21	44.09	4	1.06 (0.92–1.21)	0.45	0.00
*No*	1	1.3 (0.9–1.9)	-	-	0	-	-	-	0	-	-	-
Physical activity				12.43				58.93				0.00
*Yes*	3	1.09 (0.99–1.19)	0.07	27.55	2	1.15 (0.87–1.53)	0.32	74.26	2	1.04 (0.90–1.21)	0.58	0.00
*No*	3	1.29 (0.99–1.68)	0.06	0.00	2	1.22 (0.68–2.18)	0.51	0.00	2	1.23 (0.74–2.07)	0.43	0.00
Dietary fibre				15.92			-	-			-	-
*Yes*	5	1.10 (1.01–1.20)	**0.04**	15.92	4	1.16 (0.92–1.46)	0.21	44.09	4	1.06 (0.92–1.21)	0.45	0.00
*No*	1	1.3 (0.9–1.9)	-	-	0	-	-	-	0	-	-	-

N number of studies included in the analysis, RR relative risk, CI confidence interval, P p-value, I^2^ I-squared statistic

## Discussion

The current study provides robust evidence for positive associations between red meat consumption and CRC risk, with notable differences among meat types and cancer subtypes. Beef demonstrated the strongest and most consistent association (*RR* = 1.30, 95% *CI*: 1.10–1.54), followed by pork (*RR* = 1.17, 95% *CI*: 1.09–1.25) and lamb (*RR* = 1.11, 95% *CI*: 1.02–1.21). These findings suggest that the level of risk varies depending on the type of red meat consumed. The results align with prior research, including Chan et al., who reported an increased risk of CRC with each 100 g/day increase in red meat intake [44], and other systematic reviews that identified a 13% increased risk of CC specifically [45].

Consistent patterns emerged across all three meat types. For both beef and lamb, studies published before 2015 showed stronger associations compared to more recent studies. This trend may be linked to the 2015 IARC guidelines by the World Health Organization (WHO), which classified red meat as a Group 2 A carcinogen, indicating it is “probably carcinogenic to humans” [46]. The publication of these guidelines likely influenced subsequent research by raising awareness, improving control for confounders, altering consumption patterns, reducing publication bias, and driving methodological refinements [47–49]. However, the limited number of studies on lamb (*n* = 6) precludes definitive conclusions, warranting caution in interpreting this trend. In contrast, pork showed an opposing trend, with stronger associations observed in more recent studies. This discrepancy could reflect evolving consumption patterns or methodological improvements specific to pork-related research.

For beef and pork, case–control studies reported stronger risk estimates compared to cohort studies across all cancer types, highlighting the critical importance of study design in examining diet-cancer relationships. This discrepancy may be attributed to recall bias, a common limitation in case–control study designs [50]. Participants with CRC may disproportionately report higher beef consumption due to heightened dietary awareness after diagnosis, potentially inflating the observed association. In contrast, cohort studies collect dietary data prior to disease onset, thereby reducing the risk of recall bias and providing more reliable risk estimates. For lamb, cohort studies identified a higher risk than case–control studies, but this finding is unreliable due to the very limited data (only one cohort study versus three case–control studies).

### Beef Consumption

The significant positive association between beef consumption and CRC risk aligns with previous research suggesting that red meat consumption may increase the CRC risk [45, 51, 52]. Geographical differences, particularly pronounced for RC, suggest potential variations in the beef-CRC relationship across different populations. These differences might be attributed to variations in beef preparation methods, overall dietary patterns, or genetic factors across different regions [53–55]. For instance, the pronounced association in South American studies (*RR* = 1.47) demands further investigation and may provide insights into specific dietary or lifestyle factors that interact with beef consumption to influence cancer risk. Regional disparities in meat intake further underscore this variability, with South American studies reporting much higher median intake levels (32–236.5 g/day) compared to Asian studies (7.4–46.05 g/day).

The most consistent finding was the significant impact of adjusting for physical activity on the beef-CRC relationship. Both subgroup analyses and meta-regression consistently showed that studies not adjusted for physical activity reported stronger associations. This underscores the critical importance of accounting for physical activity in future studies examining this relationship [56]. The mechanisms underlying the potential interaction between beef consumption, physical activity, and CRC risk warrant further investigation. Physical activity may mitigate the potentially harmful effects of beef consumption through various pathways, including improved gut motility, enhanced immune function, and reduced inflammation [57–59]. Alternatively, physical activity might serve as a proxy for an overall healthier lifestyle, which could confound the observed association between beef consumption and CRC risk. Further research is needed to explore these mechanisms and their implications.

Emerging evidence suggests that microbial activities may play a role in CRC carcinogenesis, with high-protein meats providing an abundant source of nutrients that support these microbial processes. This is particularly relevant for beef consumption, which contains higher protein content compared to other red meats, according to the FAO food composition tables. The microbial fermentation of protein produces potentially harmful metabolites, and recent studies have demonstrated significant shifts in gut microbiota composition following beef consumption. Specifically, studies have observed increases in *Clostridium perfringens* species and decreases in beneficial bacteria such as *Bifidobacterium* genus after 48 h of protein fermentation from cooked beef [60].

### Pork Consumption

Our analysis of pork consumption showed weaker CRC risk associations compared to beef. This difference might be explained by pork’s lower heme iron content (heme iron in pork 11.2 mg/kg) compared to beef 28.3 mg/kg) [61]. This correlation also supports the hypothesized role of heme iron in CRC carcinogenesis through NOC formation and lipid peroxidation [62]. The moderate heterogeneity observed across analyses (*CRC*: *I*^2^ = 40.42%; *CC*: *I*^2^ = 46.59%; *RC*: *I*^2^ = 40.62%) reflects variability in effect sizes between studies, a common occurrence in dietary meta-analyses due to differences in study designs, populations, and methodologies [63, 64].

However, the consistent positive association across various subgroups for CRC strengthens the reliability of our findings. The significant impact of adjusting for physical activity and dietary fibre, particularly for RC, highlights the importance of considering these factors in future studies. The meta-regression analyses provided additional insights into the factors influencing this relationship between pork consumption and cancer risk. For CRC, the significant positive effect of dietary fibre adjustment suggests that studies incorporating this factor tend to show stronger associations. These findings suggest that the relationship between pork consumption and CRC risk may be modulated by overall lifestyles and dietary patterns [7, 65].

High-temperature cooking methods often used for pork can generate HCA and PAH—known carcinogens—which may also contribute to the observed risks [66]. The significant association observed for overall CRC compared to CC or RC alone suggests that pork consumption may potentially have a distinct effect that needs further investigation. These variations might reflect different susceptibilities to dietary factors along the colorectum, or they could be due to limitations in statistical power for site-specific analyses. Future research should focus on elucidating the potential mechanisms underlying these relationships and their variations across different colorectal sites.

### Lamb Consumption

The observed 11% increase in CRC risk associated with lamb consumption is statistically significant, though it is based on a limited number of studies (*n* = 6). While this finding is consistent with broader evidence linking red meat intake to cancer risk, there is limited data specific to lamb. This gap is especially evident for colon cancer (*n* = 4) and rectal cancer (*n* = 4), where the associations were not statistically significant. Future research should prioritise prospective cohort designs to clarify the role of lamb in CRC risk, particularly given its unique nutritional profile compared to beef and pork.

Importantly, the difference in association strength between studies that adjusted for BMI and those that did not underscores the relevance of body composition in diet-cancer associations [67]. This is consistent with our meta-regression findings for CC (*n* = 4), which indicated a significant moderating effect of BMI adjustment. Prior research has similarly reported positive associations between BMI and colorectal adenoma risk, even in metabolically healthy individuals [68, 69]. These findings reinforce the importance of accounting for BMI as a potential confounder in future research examining red meat consumption and CRC risk. Other lifestyle factors did not significantly moderate the associations in our analysis, although the small number of studies limits the strength of these conclusions.

Our meta-analysis, while comprehensive, has several important limitations. The primary limitations include the heavy reliance on self-reported dietary intake and introducing potential recall bias, particularly in case–control studies, which include 66.7% of the studies analysed. While FFQs were the most common method, the number of questions and level of detail differed significantly. Some FFQs included detailed questions on portion sizes, cooking methods, or specific cuts of meat, whereas others assessed intake in broader categories. This inconsistency may have introduced measurement error, as studies with fewer or less-specific questions could underestimate true intake or misclassify exposure levels. Another limitation is the high heterogeneity, particularly in beef consumption analyses (*I*^2^ > 70%). This suggests substantial variability in effect estimates across studies and may partially explain the observed geographical variations, as region-specific FFQs often reflect local dietary habits rather than standardised criteria. Despite our inclusion of studies from multiple continents, there was limited representation from African and Asian populations. Furthermore, while most studies adjusted for basic demographic factors, the inconsistent reporting of important lifestyle factors such as physical activity, smoking, and alcohol consumption presents another limitation. The narrower scope of our review reflects the need to distinguish between the effects of red meat and processed meat, given their distinct carcinogenic mechanisms. Our findings provide critical insights into the role of red meat as a probable risk factor, independent of processed meat.

In conclusion, our meta-analysis provides a strong positive association between red meat consumption and CRC risk, with notable variations across meat types and cancer subtypes. Beef consumption demonstrated the strongest association with CRC risk, followed by pork, while lamb consumption showed a weaker relationship. The findings underscore the complex nature of diet-cancer relationships, revealing that the association is modified by factors such as physical activity, dietary patterns, and potentially cultural differences in meat preparation. While complete meat avoidance is unnecessary, our results support current dietary guidelines recommending limited red meat consumption. Future research should focus on explaining the mechanisms underlying these associations, exploring potential effect modifiers, and investigating strategies to mitigate the risks. Key areas for investigation include the cooking and processing methods, the interaction of dietary patterns with red meat intake, and the potential protective effects of other dietary components consumed concurrently with red meat. Future studies should use standardised dietary tools to quantify red meat types and cooking methods, investigate gene-diet interactions to identify susceptible subgroups, and prioritise cohort studies in under-researched regions to account for dietary diversity. Such insights could inform more targeted dietary recommendations to reduce CRC risk.

## Supplementary Information

Below is the link to the electronic supplementary material.

ESM 1 (DOCX 914 KB)

## Data Availability

Data is provided within the manuscript or supplementary information files

## Declarations

Ethics Approval and Consent to Participate.

Not applicable.
