# Supplementary material for: Systematic Analysis of the Differential Effects of Red Meat on Colorectal Cancer Risks: A Meta-Analytic Approach
Source: J Gastrointest Cancer. 2025 Aug 7;56(1):170. doi: 10.1007/s12029-025-01247-3 (PMC12331815; doi:10.1007/s12029-025-01247-3)

Supplementary material to Woon Jun Yu, Gihani Vidanapathirana, Alfred K. Lam, Vinod Gopalan. Systematic analysis of the differential effects of red meat on colorectal cancer risks: a meta-analytic approach.

Journal of Gastrointestinal Cancer

Corresponding Authors*

**Associate Professor Vinod Gopalan**

School of Medicine & Dentistry, Griffith University, Gold Coast Campus, Gold Coast QLD 4222, Australia. E-mail: [v.gopalan@griffith.edu.au](mailto:v.gopalan@griffith.edu.au)

Telephone +61 7 56780717; Fax +61 7 56780708

**Professor Alfred K Lam**

Head of Pathology, School of Medicine & Dentistry, Gold Coast Campus, Gold Coast QLD 4222, Australia. E-mail: [a.lam@griffith.edu.au](mailto:a.lam@griffith.edu.au)

Telephone +61 7 56780718; Fax +61 7 56780303

Supplementary tables [posted as supplied by author] **Supplementary Table 1**. Summary of Inclusion Studies

| Study ID | Meat Type | Country | Study Duration | Study Design | Adjustment factors | Age (years) | Sex | Total sample size | Case | Control | Adjusted RR (95% CI) | Dietary assessment | Measure methods | Risk of Bias | | | | | | | | |
| --- | --- | --- | --- | --- | --- | --- | --- | --- | --- | --- | --- | --- | --- | --- | --- | --- | --- | --- | --- | --- | --- | --- |
|  |  |  |  |  |  |  |  |  |  |  |  |  |  | **A** | **B** | **C** | **D** | **E** | **F** | **G** | **H** | **I** |
| Sandler 1993 | Beef | USA | 3 | Case-control study | Age, BMI, alcohol consumption, and calorie intake | ≥30 | Male and Female | 645 | 236 | 409 | Male-CRC 2.07 (0.82-5.19),  Female-CRC 1.59 (0.72-3.50) | Validated FFQ | Frequency (serving/week) | + | - | + | + | + | + | + | + | - |
| Haile 1997 | Beef | USA | 2 | Case-control study | Age, gender, NSAIDs use, fat, vegetable, protein, carbohydrates, fiber, cholesterol, BMI, physical activity, calories, smoking, ethnicity | 50-74 | 66.6% male and 33.4% female | 976 | 488 | 488 | CRC 1.83 (1.12-2.99) | Validated FFQ | Portion  (g/week) | + | + | + | + | + | + | + | + | + |
| LeMarchand 1997 | Pork | USA | 4 | Case-control study | Age, BMI, family history of CRC, pack-years of smoking, alcohol consumption, dietary intake (calorie, fibre, calcium), and physical activity level | ≥18 | 58.6% male and 41.4% female | 2,384 | 1,192 | 1,192 | Male-CRC 1.2 (0.8-1.9),  Female-CRC 0.7 (0.4-1.2) | Validated FFQ | Portion  (g/day) | + | + | + | + | + | + | + | + | - |
| Boutron-Ruault 1999 | Beef, Pork, Lamb | France | 5 | Case-control study | Age, gender, and calorie intake | 30-79 | 55.8% male and 44.2% female | 480 | 171 | 309 | Beef: 1.4 (0.8-2.4); Pork: 1.0 (0.6-2.8); Lamb: 1.3 (0.9-1.9) | Validated FFQ | Portion  (g/day) | + | + | + | + | + | + | + | + | - |
| Breuer-Katschinski 2001 | Beef | Germany | 2 | Case-control study | Social class (HMSO), BMI, and total energy intake | 63.4/64.2/63.8 | 50.6% male and 49.4% female | 360 | 182 | 178 | CRC 3.10 (1.46-6.43) | Validated FFQ | Portion  (g/day) | + | + | + | + | + | + | + | + | - |
| Navarro 2003 | Beef (fatty), Beef (lean), Pork | Argentina | 5 | Case-control study | Age, gender, BMI, social status, energy intake, and dietary intake (lipids, proteins, cholesterol, fatty acids, soluble and insoluble fiber) | 23-80 | 55.5% male and 44.5% female | 851 | 287 | 564 | Beef (fatty): CRC 0.78 (0.51-1.18);  Beef (lean): CRC 0.67 (0.40-0.97);  Pork: CRC 0.92 (0.62-1.36) | Validated FFQ | Portion  (g/day) | + | + | + | + | + | + | + | + | ? |
| Brink 2005 | Beef, Pork | Netherlands | 8 | Prospective cohort study | Age, gender, Quetelet Index (QI), family history of CRC, smoking status, and energy intake | 55-69 | 48.4% male and 51.6% females | 608 | 182 | 426 | Beef: CC 1.28 (0.96-1.72),  RC 0.92 (0.57-1.49);  Pork: CC 0.77 (0.57-1.04),  RC 0.70 (0.43-1.13) | Validated FFQ | Portion  (g/day) | + | + | + | + | + | + | + | - | + |
| Lüchtenborg 2005 | Beef, Pork | Netherlands | 8 | Prospective cohort study | Age, gender, BMI, family history of CRC, smoking status, and total energy intake | 55-69 | 48% male and 52% female | 2,948 | 588 | 2,360 | Beef: CC 1.29 (0.96-1.73),  RC 0.95 (0.59-1.54);  Pork: CC 0.77 (0.57-1.04),  RC 0.70 (0.44-1.13) | Validated FFQ | Portion  (g/day) | + | + | + | + | + | + | + | - | + |
| Norat 2005 | Beef, Pork, Lamb | European Countries | 6 | Prospective cohort study | Age, gender, height, weight, study centre location, smoking status, alcohol consumption, energy from non-fat sources, energy from fat sources, dietary fibre intake, and physical activity level | 21-83 | 29.7% male and 70.3% female | 478,040 | 1,329 | 476,711 | Beef: CRC 1.03 (0.86-1.24);  Pork: CRC 1.18 (0.95-1.48);  Lamb: CRC 1.22 (0.96-1.55) | Validated FFQ | Portion  (g/day) | + | + | + | + | + | + | + | - | + |
| Aune 2009 | Beef, Lamb | Uruguay | 8 | Case-control study | Age, gender, education level, residence area, income, interviewer, smoking status, cigarettes per day, age at initiation of smoking, years since quitting smoking (for former smokers), duration of smoking, BMI, alcohol consumption, mate drinking, energy intake, and dietary intake (grains, vegetables, fruits, fatty foods, dairy, fish, poultry, processed meat) | 26-89 | 62.3% male and 37.7% female | 2,032 | 722 | 1,310 | Beef: CRC 3.36 (2.08-5.42),  CC 3.13 (1.57-6.26),  RC 3.32 (1.83-6.02);  Lamb: CRC 1.22 (0.68-2.21),  CC 0.87 (0.35-2.18),  RC 1.35 (0.66-2.77) | Non-validated FFQ | Portion  (g/day) | + | + | + | + | + | + | - | + | + |
| Aune 2009 | Beef, Lamb | Uruguay | 12 | Case-control study | Age, gender, education level, residence area, smoking status, cigarettes per day, age at initiation of smoking, years since quitting smoking (for former smokers), duration of smoking, type of tobacco used, alcohol consumption, and dietary intake (vegetables, fruits, milk) | 20-89 | 50.8% male and 49.2% female | 8,724 | 6,892 | 1,832 | Beef: CRC 2.04 (1.47-2.83),  CC 2.00 (1.19-3.35),  RC 1.92 (1.18-2.00);  Lamb: CRC 1.32 (0.82-2.13),  CC 1.53 (0.72-3.26),  RC 1.12 (0.53-2.35) | Non-validated FFQ | Frequency  (times/week) | + | + | + | + | + | + | - | + | + |
| Nayak 2009 | Beef | India | 3 | Case-control study | Age, gender, medical history, sociodemographic characteristics, smoking status, and alcohol consumption | 18-85 | 70.4% male and 29.6% female | 432 | 108 | 324 | CRC 4.25 (2.02-8.94) | FFQ (no validation mentioned) | Frequency (times/week) | + | + | + | + | + | + | ? | + | ? |
| Fu 2011 | Beef, Pork | USA | 7 | Case-control study | Age, gender, race, BMI, education level, study centre location, recruitment timing, colonoscopy indication, smoking status, alcohol consumption, total energy intake, NSAIDs usage, and physical activity level | 40-75 | 61.3% male and 38.7% female | 6,307 | 2,543 | 3,764 | Beef: 1.3 (1.1-1.5);  Pork: 1.4 (1.2-1.6) | Validated FFQ | Portion (g/day) | + | + | + | + | + | + | + | + | - |
| Tantamango 2011 | Beef | USA | 28 | Prospective cohort study | Age, gender, and BMI | 73.4/71.2 | 40.2% male and 59.8% female | 2,818 | 441 | 2,377 | 1.09 (0.84-1.41) | FFQ (no validation mentioned) | Frequency (times/week) | - | + | ? | + | + | + | + | + | + |
| Egeberg 2013 | Beef, Pork, Lamb, Veal | Denmark | 16 | Prospective cohort study | Waist circumference, education level, smoking status, alcohol consumption, total energy intake, dietary fibre intake, NSAIDs usage, hormone replacement therapy use, and physical activity level | 50-64 | 47.8% male and 52.2% female | 53,988 | 989 | 52,999 | Beef: CC 1.30 (1.00-1.70),  RC 0.75 (0.52-1.09);  Pork: CC 1.04 (0.79-1.37),  RC 1.63 (1.11-2.39);  Lamb: CC 1.35 (1.07-1.71),  RC 1.12 (0.83-1.53);  Veal: CC 1.01 (0.79-1.28),  RC 1.18 (0.85-1.64) | Validated FFQ | Portion (g/day) | + | + | + | + | + | + | + | + | + |
| Gilsing 2015 | Beef, Pork | Netherlands | 20.3 | Prospective cohort study | Age, gender, education level, BMI, smoking status, alcohol consumption, total energy intake, and physical activity level | 55-69 | 47.5% male and 52.5% female | 10,609 | 836 | 9,773 | Beef: CRC 1.04 (0.77-1.40),  CC 1.09 (0.77-1.54),  RC 0.72 (0.35-1.48);  Pork: CRC 1.14 (0.80-1.62),  CC 0.88 (0.58-1.33),  RC 2.08 (0.88-4.91) | Validated FFQ | Portion (g/day) | + | + | + | + | + | + | + | + | + |
| Joshi 2015 | Beef, Pork | Canada and USA | - | Case-control study | Age, gender, race, study center location, BMI, dietary intake (calorie, fiber, vegetables, saturated fat), and physical activity levels | 59.4 | 46% male and 54% female | 6,854 | 3,350 | 3,504 | Beef: CRC 1.1 (0.9-1.3),  CC 1.0 (0.8-1.2),  RC 1.2 (0.9-1.6);  Pork: CRC 1.1 (1.0-1.3),  CC 1.1 (0.9-1.3),  RC 1.1 (0.9-1.5) | Validated FFQ | Portion (g/1000 kcal/day) | + | + | + | + | + | + | + | + | - |
| Angelo 2016 | Beef, Pork | Brazil | - | Case-control study | Age, gender, ethnicity, smoking status, and alcohol consumption | 29-87 | 64.4% male and 35.6% female | 270 | 169 | 101 | Beef: 1.025 (1.007-1.044);  Pork: 1.121 (1.061-1.185) | Validated FFQ | Portion (120g/month) | + | - | + | + | + | + | + | + | ? |
| Vulcan 2017 | Beef, Pork | Sweden | 19 | Prospective cohort study | Age, gender, method version, season, education level, smoking status, alcohol consumption, total energy intake, non-steroidal anti-inflammatory drugs (NSAIDs) usage, and physical activity level | 50-69 | 39.3% male and 60.7% female | 27,931 | 728 | 27,203 | Beef: CRC 0.79 (0.62-1.01),  CC 0.60 (0.44-0.82),  RC 1.23 (0.83-1.83);  Pork: CRC 1.39 (1.09-1.78),  CC 1.41 (1.04-1.90),  RC 1.47 (0.47-2.44) | Validated FFQ | Portion (g/day) | + | + | + | + | + | + | + | + | + |
| Wang 2018 | Pork | China | 3 | Case-control study | Age, gender, marital status, occupation, education level, BMI, depression, psychosis, nearby factory pollution, alcohol consumption, dietary intake (fibres, garlic, fried food, barbecued meat), and physical activity level | 33-83 | 45.9% male and 54.1% female | 634 | 317 | 317 | CRC 1.587 (1.044-2.414) | FFQ (internally validated) | Frequecy | + | ? | + | + | + | + | + | + | ? |
| Islam 2019a | Beef, Pork | Japan | 18 | Population-based cohort study | Age, residence area, BMI, history of diabetes, smoking status, alcohol consumption, log-transformed energy intake, dietary intake (fibre and calcium), and physical activity level | ≥35-74 | Male and Female | 127,185 | 3,550 | 123,635 | Beef: Male-CRC1.08 (0.93-1.26), CC 1.06 (0.92-1.24), RC 1.08 (0.83-1.41), Female-CRC 1.09 (0.94-1.28), CC 1.20 (1.01-1.44), RC 0.95 (0.71-1.28); Pork: Male-CRC 1.11 (0.82-1.49), CC 1.05 (0.80-1.38), RC 1.16 (0.88-1.55), Female-CRC 1.04 (0.89-1.22), CC 1.04 (0.86-1.25), RC 1.04 (0.78-1.39) | Validated FFQ | Portion (per 50/100 g) | + | + | + | + | + | + | + | + | + |
| Islam 2019b | Beef, Pork | Japan | 18 | Population-based cohort study | Age, residence area, BMI, history of diabetes, smoking status, alcohol consumption, log-transformed energy intake, dietary intake (fibre and calcium), and physical activity level | 40-79 | Male and Female | 232,403 | 3,550 | 228,853 | Beef: Male-CRC 1.05 (0.68-1.63), CC 1.10 (0.61-1.97), RC 1.32 (0.68-2.56), Female-CRC 1.19 (0.67-2.12), CC 1.16 (0.55-2.47), RC 2.12 (0.86-5.18); Pork: Male-CRC 0.98 (0.77-1.26), CC 0.99 (0.68-1.43), RC 1.05 (0.72-1.53), Female-CRC 1.03 (0.75-1.41), CC 0.91 (0.62-1.35), RC 1.38 (0.81-2.35) | Validated FFQ | Frequency (times/week) | + | + | + | + | + | + | + | + | + |
| Saliba 2019 | Beef, Pork, Lamb | Israel | - | Case-control study | Age, gender, education level, BMI, family history of CRC, smoking status, alcohol consumption, total energy intake, dietary intake (fibre, calcium, vegetables, fruits, vitamin D, folate), regular aspirin intake, and physical activity level | 71.7/70.1; 64.0/61.0 | 52.6% male and 47.4% female | Jews: 8,615; Arab: 1,411 | 4,615 | Jews: 4,000; Arabs: 554 | Beef: Jews-CRC1.01 (0.97-1.05), Arabs-CRC 0.88 (0.81-0.96); Pork: Jews-CRC 1.21 (1.07-1.35), Arabs-CRC 1.15 (0.89-1.48); Lamb: Jews-CRC 1.46 (1.20-1.79), Arabs-CRC 1.02 (0.91-1.16) | Validated FFQ | Frequency (serving/week) | + | - | + | + | + | + | + | + | + |
| Deoula 2020 | Beef, Lamb | Morocco | 8 | Case-control study | Age, education level, residence area, monthly income, smoking status, BMI, family history of CRC, NSAIDs usage, alcohol consumption, total energy intake, dietary intake (fibre, vegetables, fruits, calcium, dairy, fish), and physical activity level | ≥18 | 49.3% male and 50.7% female | 2,906 | 1,453 | 1,453 | Beef: CRC 1.11 (1.01-1.24), CC 1.23 (1.05-1.44), RC 1.01 (0.86-1.17); Lamb: CRC 1.01 (0.90-1.14), CC 1.01 (0.85-1.19), RC 1.02 (0.86-1.20) | Validated FFQ | Portion (g/week) | + | + | + | + | + | + | + | + | + |
| Mehta 2020 | Beef, Pork | USA | 12.7 | Prospective cohort study | Race/ethnicity, education level, BMI, family history of CRC, calorie intake, and physical activity level | 35-74 | Female | 48,704 | 216 | 48,488 | Beef: CRC 1.18 (0.82-1.9); Pork: CRC 1.25 (0.85-1.84) | Validated FFQ | Portion (g/day) | - | + | - | + | + | + | + | - | + |
| Ramírez-Díaz 2021 | Beef, Pork | Mexico | <1 | Case-control study | Residence area, marital status, physical activity level, and dietary intake (fruits, beef, pork, fried foods) | >20 | 59.2% male and 40.8% female | 294 | 98 | 196 | Beef: CRC 2.95 (1.05-8.26); Pork: CRC 3.26 (1.34-7.90) | Validated FFQ | Frequency (times/week) | + | + | + | + | + | + | + | + | ? |
| Tee 2023 | Pork | Malaysia | <1 | Case-control study | Age, gender, race, education level, medical history, dietary intake (pork, preserved foods), and physical activity | 40-75 | 43.1% male and 56.9% female | 757 | 148 | 609 | CRC 2.29 (1.09-4.79) | Validated FFQ | Frequency (times/week) | + | + | + | + | + | + | + | + | ? |
| Wismayer 2024 | Beef | Uganda | 2 | Case-control study | Residence area, BMI, CRC status, smoking status, and alcohol consumption | 42-67.5 | 50% male and 50% female | 384 | 128 | 256 | CRC 1.73 (0.45-6.72) | Validated FFQ | Frequency (times/week) | + | + | + | + | + | + | + | + | ? |

***Note:***

**Risk of Bias in Cohort Studies/Case-Control Studies**

**A**: Representativeness: Population-based cohort/Case Definition: Histologically confirmed CRC

**B**: Non-Exposed: Drawn from the same population as exposed/Representativeness: Multi-centre recruitment (>2 hospitals) or from the largest hospital in the area

**C**: Exposed Ascertainment: Validated tool (e.g., FFQ)/Control Selection: Population-based or same source as cases

**D**: Outcome Absence at Baseline: No prior history of the outcome (CRC)/Control Definition: No CRC history

**E**: Control of Age/Sex

**F**: Control for ≥1 Additional Confounder (e.g., BMI, smoking, diet)

**G**: Outcome Assessment: Independent blind review or registry linkage/Exposure Ascertainment: Validated FFQ

**H**: Follow-Up Duration: ≥10 years/Same Method for All: Identical tool for cases/controls

**I**: Adequacy: Loss to follow up <20%/Non-Response Rate: <20% for cases/controls

Supplementary figures [posted as supplied by author] **Supplementary Figure 1**. Contour-enhanced funnel plot of beef consumption and colorectal cancer risk.


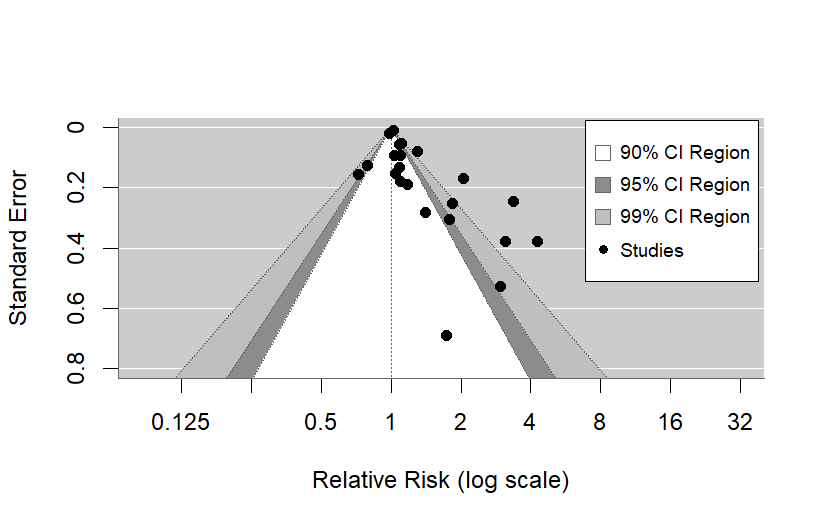


**Supplementary Figure 2**. Contour-enhanced funnel plot of beef consumption and colon cancer risk.
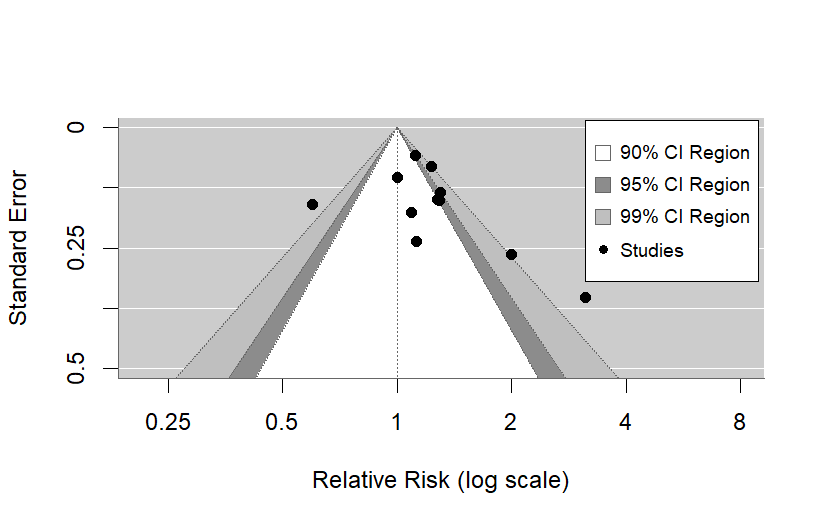


**Supplementary Figure 3**. Contour-enhanced funnel plot of beef consumption and rectal cancer risk.
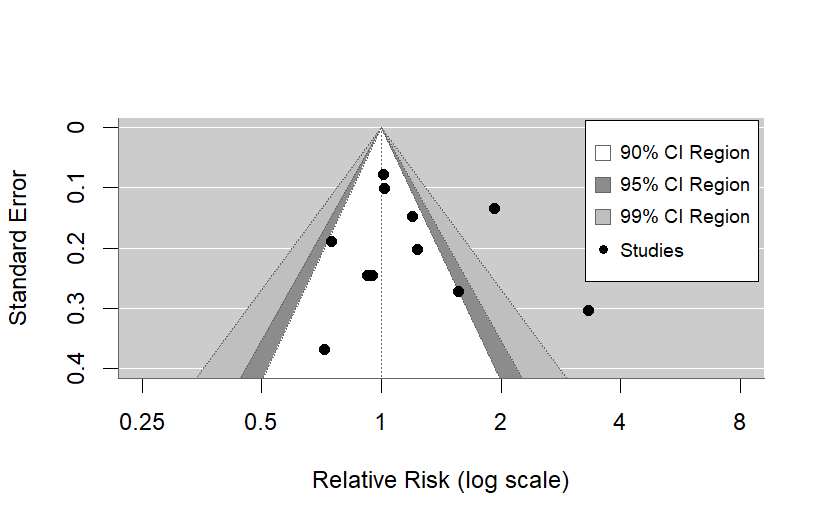


**Supplementary Figure 4**. Contour-enhanced funnel plot of pork consumption and colorectal cancer risk.
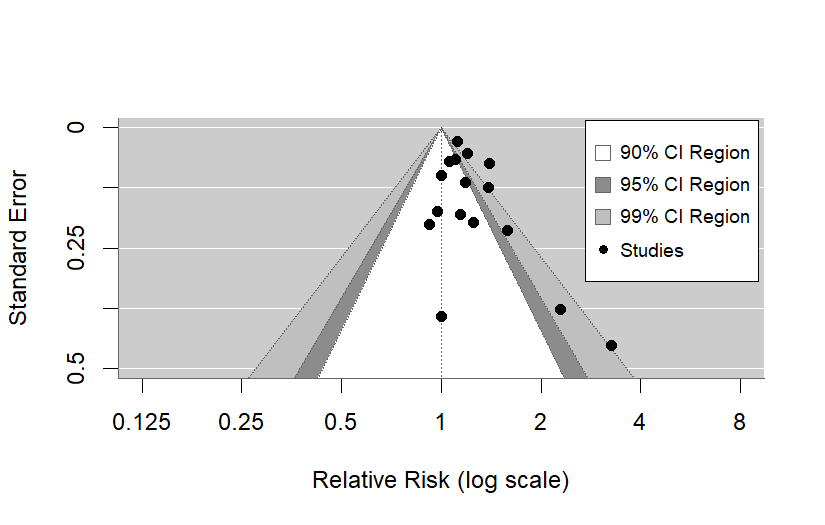


**Supplementary Figure 5**. Contour-enhanced funnel plot of pork consumption and colon cancer risk.
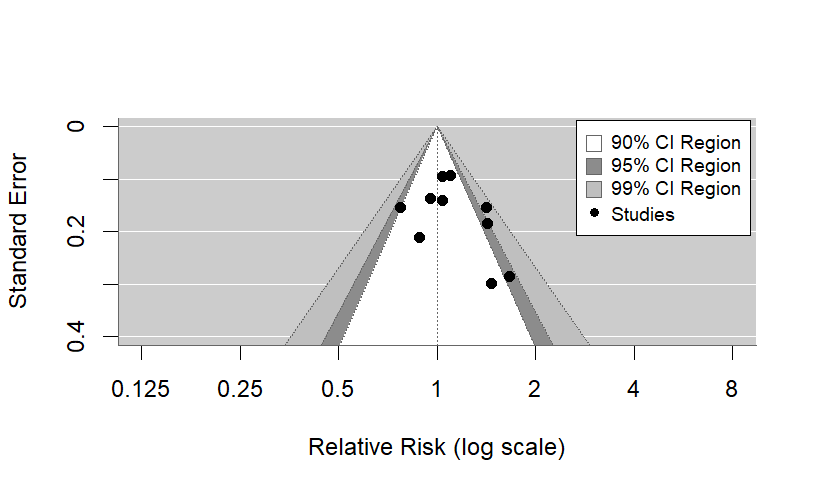


**Supplementary Figure 6**. Contour-enhanced funnel plot of pork consumption and rectal cancer risk.
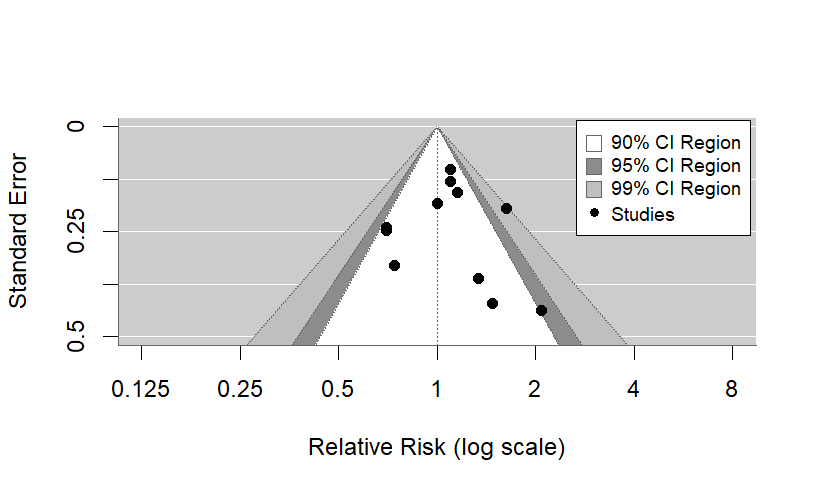


**Supplementary Figure 7**. Forest plot of cohort and case-control studies of colorectal cancer risk associated with lamb consumption.

**Supplementary Figure 7**. Forest plot of cohort and case-control studies of colorectal cancer risk associated with lamb consumption.

**
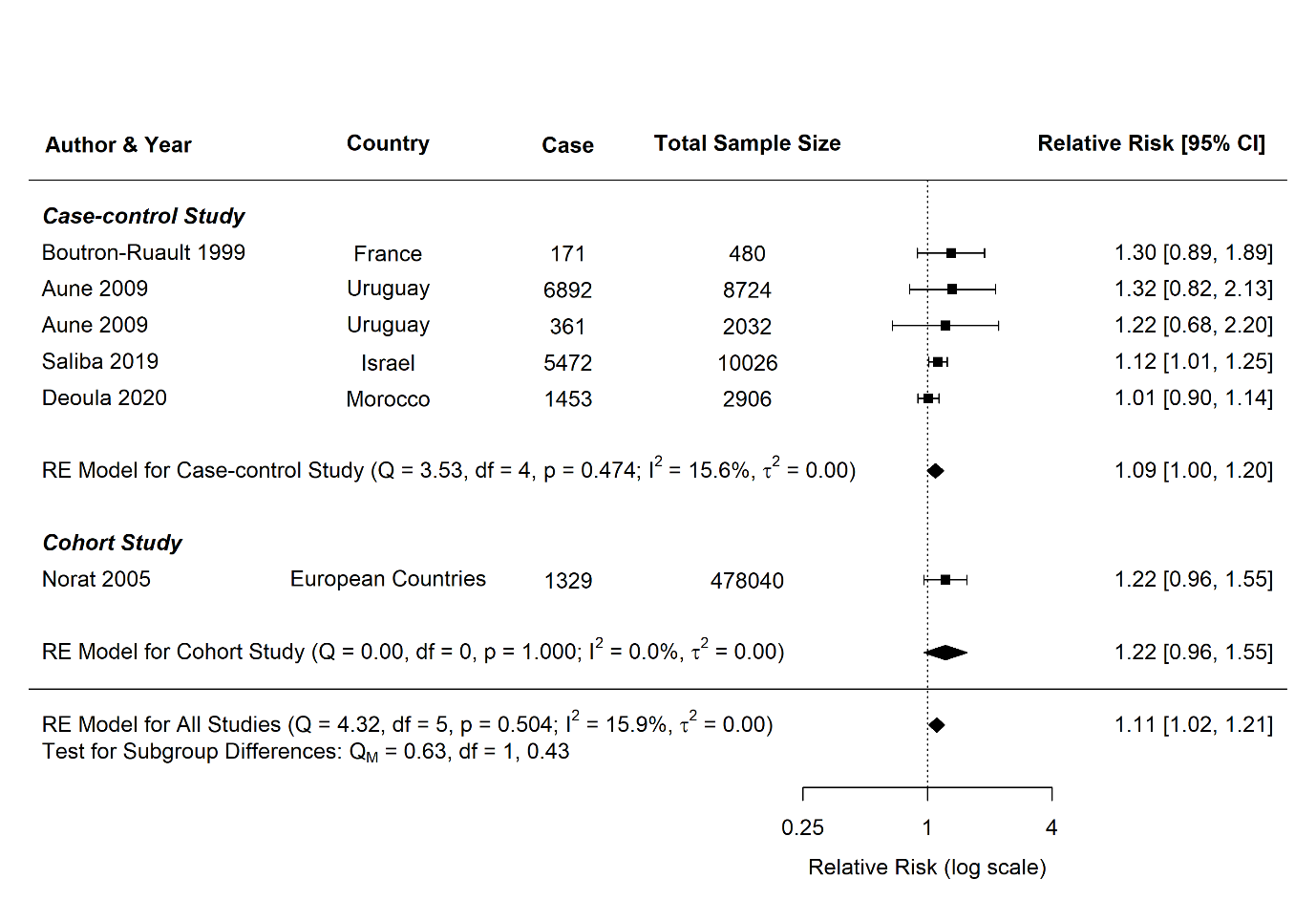
Supplementary Figure 8**. Forest plot of cohort and case-control studies of colon cancer risk associated with lamb consumption.


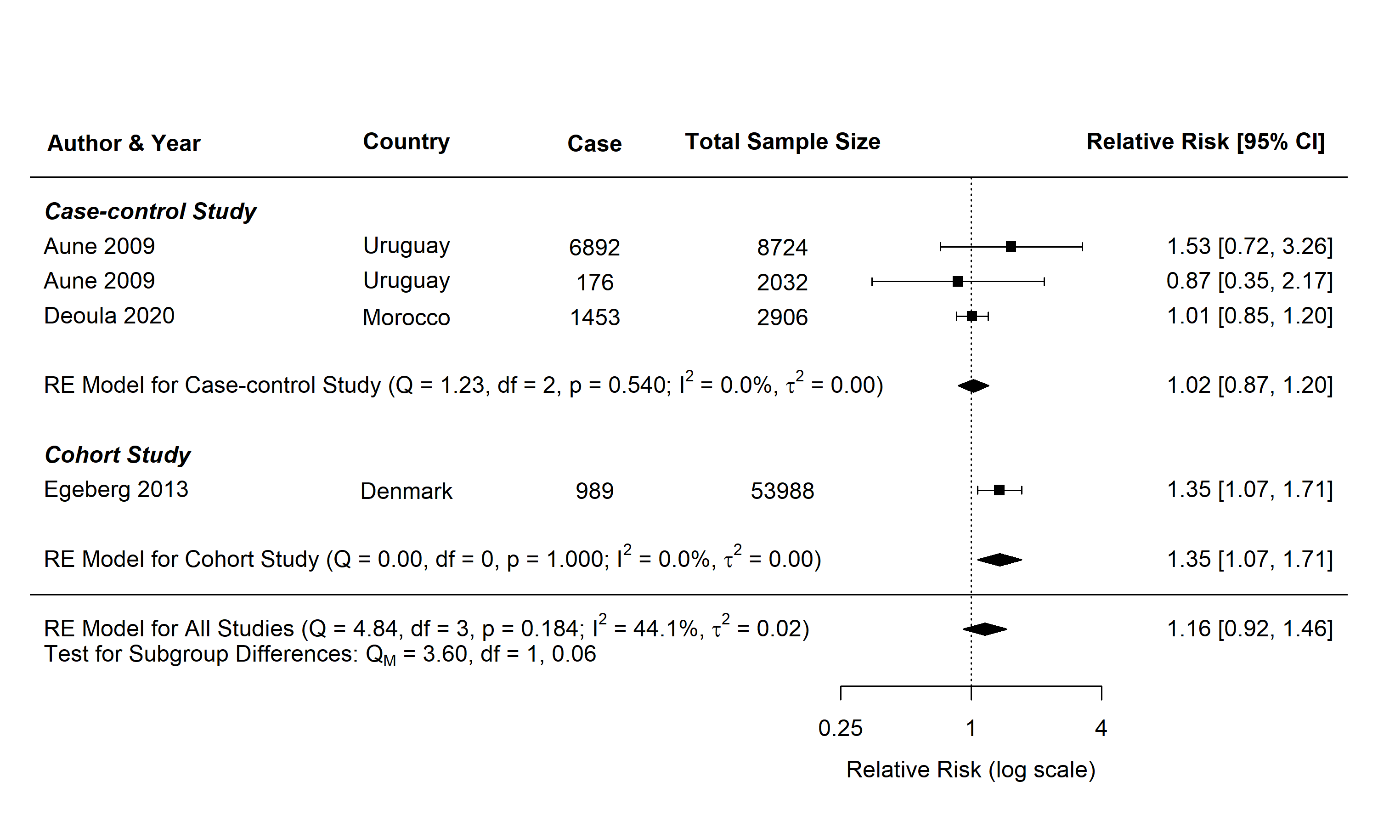


**Supplementary Figure 9**. Forest plot of cohort and case-control studies of rectal cancer risk associated with lamb consumption.


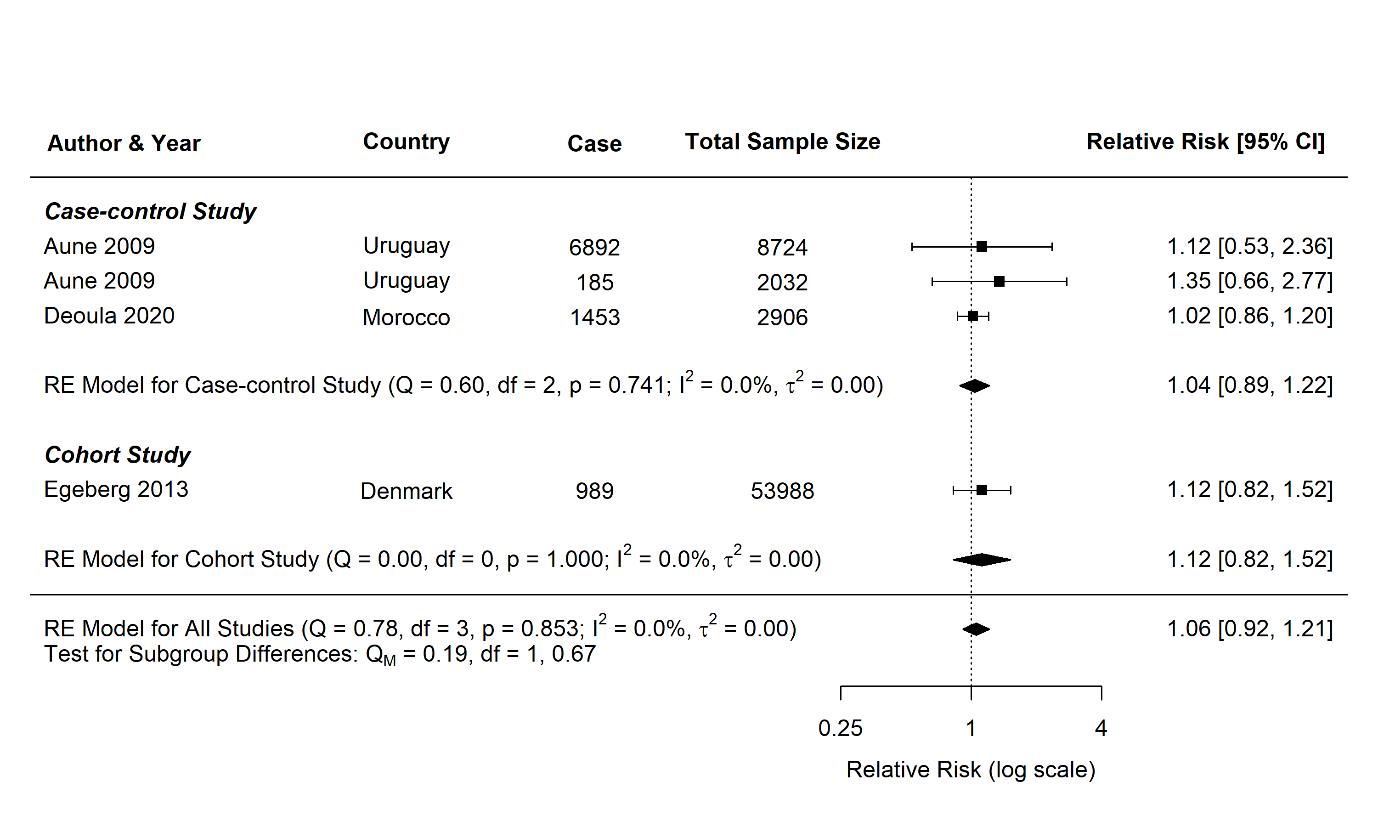


**Supplementary Figure 10**. Contour-enhanced funnel plot of lamb consumption and colorectal cancer risk.
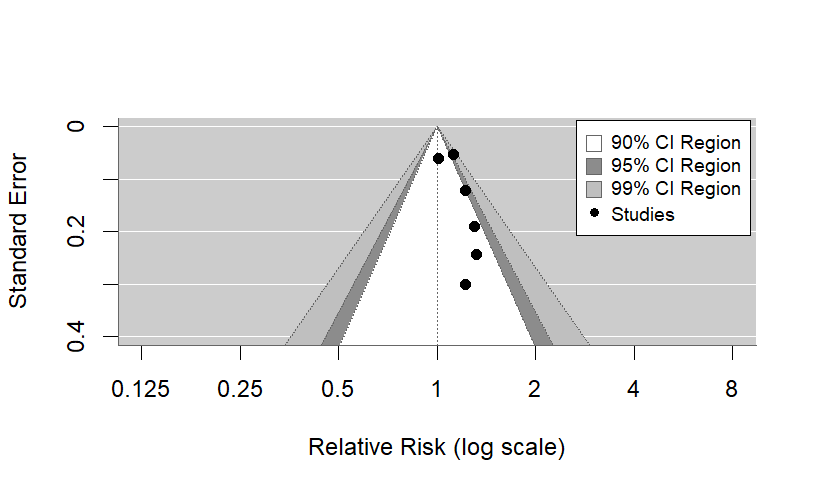


**Supplementary Figure 11**. Contour-enhanced funnel plot of lamb consumption and colon cancer risk.
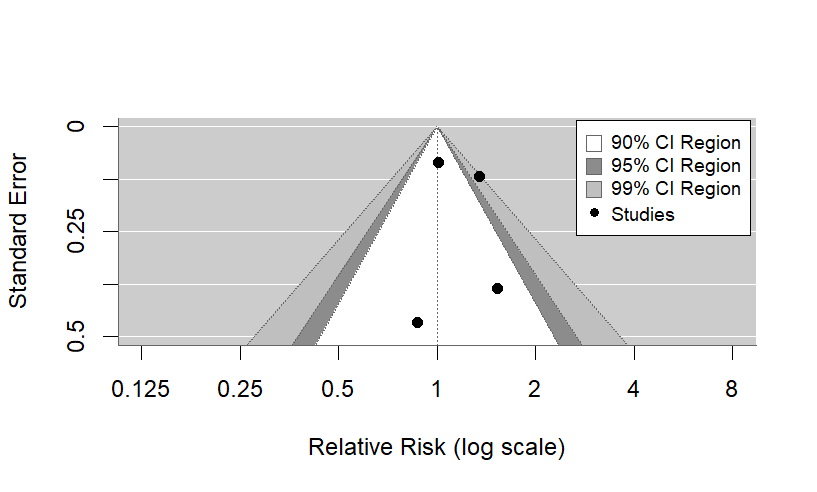


**Supplementary Figure 12**. Contour-enhanced funnel plot of lamb consumption and rectal cancer risk.
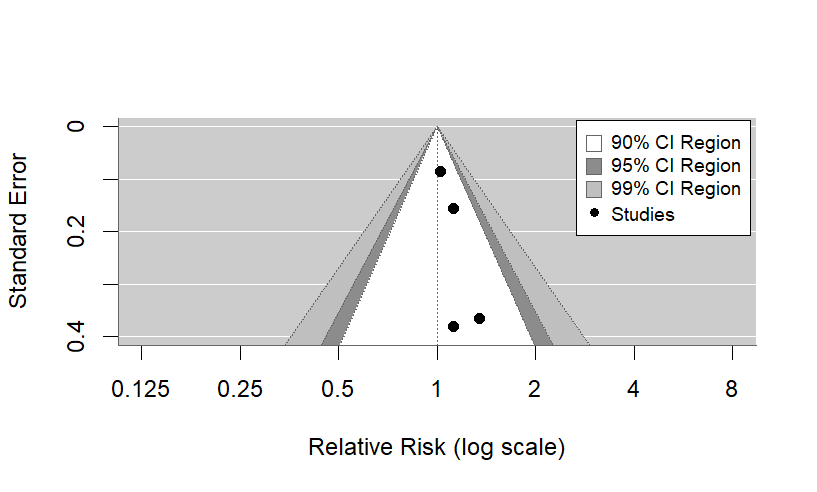

Supplement: Supplementary file 1 — (DOCX 914 KB) [file 12029_2025_1247_MOESM1_ESM.docx]
